# Detection of QTL for traits related to adaptation to sub-optimal climatic conditions in chickens

**DOI:** 10.1186/s12711-017-0314-5

**Published:** 2017-04-20

**Authors:** Ching-Yi Lien, Michèle Tixier-Boichard, Shih-Wen Wu, Woei-Fuh Wang, Chen Siang Ng, Chih-Feng Chen

**Affiliations:** 1 GABI, INRA, AgroParisTech, Université Paris-Saclay, 78350 Jouy-en-Josas, France; 2 Department of Animal Science, National Chung Hsing University, 145 Xingda Rd., South District, Taichung, 40227 Taiwan; 3 Livestock Research Institute, Council of Agriculture, Executive Yuan, 112 Muchang, Xinhua District, Tainan, 71246 Taiwan; 4 Fonghuanggu Bird and Ecology Park, National Museum of Natural Science, 1-9 Renyi Rd., Lugu Township, Nantou County, 55841 Taiwan; 5 Biodiversity Research Center, Academia Sinica, 128 Academia Rd., Section 2, Nankang, Taipei, 11529 Taiwan; 6 Institute of Molecular and Cellular Biology, National Tsing Hua University, No. 101, Section 2, Kuang-Fu Rd., Hsinchu, 30013 Taiwan; 7 Center for the Integrative and Evolutionary Galliformes Genomics, National Chung Hsing University, No. 250, Guoguang Rd., South District, Taichung, 40227 Taiwan

## Abstract

**Background:**

Growth traits can be used as indicators of adaptation to sub-optimal conditions. The current study aimed at identifying quantitative trait loci (QTL) that control performance under variable temperature conditions in chickens.

**Methods:**

An F2 population was produced by crossing the Taiwan Country chicken L2 line (selected for body weight, comb area, and egg production) with an experimental line of Rhode Island Red layer R- (selected for low residual feed consumption). A total of 844 animals were genotyped with the 60 K Illumina single nucleotide polymorphism (SNP) chip. Whole-genome interval linkage mapping and a genome-wide association study (GWAS) were performed for body weight at 0, 4, 8, 12, and 16 weeks of age, shank length at 8 weeks of age, size of comb area at 16 weeks of age, and antibody response to sheep red blood cells at 11 weeks of age (7 and 14 days after primary immunization). Relevant genes were identified based on functional annotation of candidate genes and potentially relevant SNPs were detected by comparing whole-genome sequences of several birds between the parental lines.

**Results:**

Whole-genome QTL analysis revealed 47 QTL and 714 effects associated with 178 SNPs were identified by GWAS with 5% Bonferroni genome-wide significance. Little overlap was observed between the QTL and GWAS results, with only two chromosomal regions detected by both approaches, i.e. one on GGA24 (GGA for *Gallus gallu*s chromosome) for BW04 and one on GGAZ for six growth-related traits. Based on whole-genome sequence, differences between the parental lines based on several birds were screened in the genome-wide QTL regions and in a region detected by both methods, resulting in the identification of 106 putative candidate genes with a total of 15,443 SNPs, of which 41 were missense and 1698 were not described in the dbSNP archive.

**Conclusions:**

The QTL detected in this study for growth and morphological traits likely influence adaptation of chickens to sub-tropical climate. Using whole-genome sequence data, we identified candidate SNPs for further confirmation of QTL in the F2 design. A strong QTL effect found on GGAZ underlines the importance of sex-linked inheritance for growth traits in chickens.

**Electronic supplementary material:**

The online version of this article (doi:10.1186/s12711-017-0314-5) contains supplementary material, which is available to authorized users.

## Background

Detection of quantitative trait loci (QTL) is a classical approach to better understand the genetic architecture of complex traits and unravel genomic regions that control quantitative variation of traits. Many QTL detection studies have been conducted in chickens, as reviewed by Abasht et al. [1], most of these using commercial or experimental lines, and only a few using local breeds. Since the publication of the Abasht et al. [1] review, additional QTL studies have been reported on egg laying [2, 3] and comb traits [4]. In general, these QTL studies are conducted under standard management practice. In the current study, we carried out a QTL detection project under the real conditions of a humid sub-tropical climate in Taiwan, using an F2 cross between a locally adapted line, named L2, and an imported selected line, named R-. The L2 line is a closed line that has been selected for egg production as a dam line in the mating design used for the production of Taiwan Country chicken since 1982 [5]. The R- line is a closed line that has been selected in France for adult residual food consumption since 1975 and was imported in Taiwan in 2003 [6]. This work focused on traits that are measured during the growing period and that likely play a role in the adaptation of chickens to sub-tropical climate. Body weight was shown to play an important role in adaptation to high temperatures [7, 8], shank length and comb area are non-feathered areas that contribute largely to heat dissipation [9], and antibody response to sheep red blood cells is an indicator of immune response [10]. The analysis used two different methods, genome-wide association analysis (GWAS) and QTL mapping, which were applied to genotypes obtained with the high-density 60 K Illumina iSelect chicken array [11].

## Methods

### Experimental population

A three-generation design was produced by crossing two pedigreed parental lines, L2 and R-, which are maintained at the experimental farm of the National Chung Hsing University (NCHU). Line L2 was established in 1983 from a sample of Taiwan Country chickens and has been maintained as a closed population since then [5]. L2 males were selected on the basis of the size of the comb area (as an indicator of sexual maturity), body weight at 12 or 14 weeks of age, and average egg production to 40 weeks of age of full- and half-sisters. L2 females were selected for egg production to 40 weeks of age based on their own records and those of their full- and half-sisters. Since 1975, a divergent selection was undertaken at the French National Institute for Agricultural Research (INRA) to study the genetic basis of residual feed intake (RFI) in Rhode Island Red (RIR) layers [12]. The R- line has been selected for low RFI (residual food consumption adjusted for body weight (BW), change in body weight (ΔBW) and total egg mass (EM) by multiple linear regression). Individual feed intake, BW and ΔBW, were measured between 33 and 37 weeks of age in birds of both sexes, and EM was recorded over the same period (i.e. 28 consecutive days) in females. R- males were selected on the basis of their own RFI value, whereas R- females were selected in two steps, combining their RFI value and their own egg production at 40 weeks of age. In 2003, a subset of the 28th generation of selection of R- line was shipped to NCHU to study the adaptation of R- chickens to sub-tropical climate [6]. Since then, this line has been maintained as a closed population with a one-year generation interval.

In 2009, 46 F0 parents, born in the same batch, were used to generate the F1 generation by mating six L2 males to 15 R- females (LR) and seven R- males to 18 L2 females (RL). Then, six F1 males (four LR and two RL) were mated to 51 unrelated F1 dams; 32 RL females were mated to four LR males and 19 LR females were mated to two RL males. The family size of dams ranged from 1 to 35 (see Additional file 1: Figure S1). A total of 743 F2 individuals were produced in four successive batches with the following birth dates: 17 Dec. 2010, 18 Jan. 2011, 31 Jan. 2011, and 18 Feb. 2011 (see Additional file 2: Table S1). The experimental period lasted until 16 weeks of age for each batch.

### Husbandry

All F2 chickens were reared in floor pens in an open-sided building, with a temporary fence to close the rooms, and additional heating (24 h/day) for the first two weeks (up to 29 °C). The temporary fences were removed at three weeks of age. The mean ambient temperature in the surrounding area ranged from 18 to 26 °C from batch 1 to batch 4, with minimal values ranging from 6.6 to 20 °C and maximal values ranging from 24 to 35.6 °C (see Additional file 3: Figure S2). Chicks were fed according to recommended nutrition standards, with a starter diet (metabolizable energy: 2830 kcal ME/kg and 19.14% crude protein) from hatch to 4 weeks of age and a grower diet (2818 kcal ME/kg and 16.11% crude protein) from 5 to 16 weeks of age. The same vaccination plan was applied to all F2 birds (see Additional file 4: Figure S3). Natural light was supplied during the rearing period, with an average day length close to 12 h.

### Phenotypic measurements

Body weight was measured with an electronic scale (±1 g) at hatch (BW00) and every four weeks thereafter, i.e. BW04, BW08, BW12 and BW16. Growth rate was estimated by the difference between successive measurements of body weight every 4 weeks, i.e. between 4 and 8 weeks of age (BW0804), between 8 and 12 weeks of age (BW1208), and between 12 and 16 weeks of age (BW612). Length and height of the comb at 16 weeks of age were measured with a ruler (±1 mm), and comb area was computed as the product of comb length by comb height (CA16). Length of the right shank was measured with a Vernier caliper (±1 mm) at 8 weeks of age (SL08).

All F2 chickens were injected with 0.1 mL of a 0.25% suspension of sheep red blood cell (SRBC) at 11 weeks of age. Blood was sampled from the wing vein of each bird just before immunization to determine the baseline antibody level (SRBC00) and antibody levels at 7 and 14 days after primary immunization, i.e. SRBC07 and SRBC14, respectively. Sera were collected after centrifuging the blood samples, and stored at −20 °C until all assays could be run simultaneously. SRBC antibody levels were assayed by the hemagglutination test [13] and expressed in log_2_ units. All experiments involving animals in this study were done according to the approved protocol of the Institutional Animal Care and Use Committees of the NCHU (Taichung, Taiwan; IACUC No. 97-99).

### Genotyping and quality control

At each of the three generations of the experimental design, blood was collected from the wing vein of each bird and genomic DNA was extracted using a commercial DNA extraction kit (DNeasy^®^ Blood kit) and diluted to 50 ng/μl. After DNA quality control, each chicken was genotyped using the Illumina 60 K Chicken SNP iSelect chip. Of the 846 chickens (F0: 46, F1: 57, and F2: 743), two F2 individuals were excluded because SNPs for these individuals had a low call rate (<95%). The SNP set used in the current study included 57,636 SNPs. Approximately 30% (17,018) of the SNPs did not meet the following criteria and were removed: low call rate (<95%), low minor allele frequency (<0.05), or unknown chromosomal position on the chicken reference genome GalGal4. Pedigree checking was considered necessary before performing linkage analysis. An in-house software developed by D. Boichard (personal communication) was used to detect incompatible genotypes for each sire/dam/progeny triplet. Each incompatibility with either the sire, the dam, or both was recorded. When the total number of incompatible SNPs exceeded 2% of all SNPs, another in-house software, also developed by D. Boichard (personal communication), was used to assign the most probable parent(s) to each progeny. About 10% of pedigree errors were identified and corrected. When the pedigree was confirmed, only a few incompatible SNP genotypes remained for some F2 individuals compared to their F1 parents, and these were considered as genotyping errors rather than as pedigree errors and replaced by missing values for the corresponding F2 individuals. The final data included genotypes for 844 individuals (F0, F1, and F2) and 40,618 SNPs, distributed on 28 autosomes, two linkage groups and the Z sex chromosome. The *F*_st_ between parental lines was calculated with PLINK (Version 1.9) [14] using the SNP genotypes from the 46 F0 parents.

### Phenotypic comparisons

The normality of trait distribution in the F2 population was checked by the SAS^®^ UNIVARIATE procedure (Statistical Analysis System, Version 9.3, SAS, Institute Inc., Cary, NC, USA). Trait differences between parental lines were studied by analysis of variance using the GLM procedure in SAS^®^ and including the effects of sex and line. For the F2 population, the GLM procedure was applied to estimate the fixed effects of sex, dam (full-sib family effect), and batch, taking into account BW00 as a covariate for all other body weight measurements, as well as for SL08 and CA16. For the SRBC traits, the model included BW08 instead of BW00 as a covariate, in addition to the fixed effects.

### Genome-wide association analysis

Population stratification of the F2 individuals was assessed with multidimensional scaling (MDS) analysis available from PLINK (Version 1.0.7) [15]. The indep-pairwise option with a window size of 25 SNPs, a step of 5 SNPs, and an r^2^ threshold of 0.2 was used to obtain independent SNPs. Pairwise identity-by-state (IBS) distances were calculated between all individuals using 3266 independent SNPs, and MDS components were estimated by the mds-plot option based on the IBS matrix. Linkage disequilibrium (LD) blocks were defined as a set of contiguous SNPs with pairwise r^2^ values exceeding 0.4, which resulted in 7063 LD blocks for growth-related traits and 7048 LD blocks for SRBC traits.

GWAS was carried out by using a univariate linear mixed model in GEMMA [16]. The linear mixed model used in this study was applied for each chromosome, with sex, batch, and dam as fixed effects and by incorporating random genetic effects with genomic relationships to correct for genetic structure in the experimental population. The analysis was implemented on all data for the autosomes, while data from males and females were separated for the analyses on the Z chromosome. *P* value thresholds were computed based on the number of independent SNPs and LD blocks [17, 18], resulting in a P-value threshold of 5% Bonferroni genome-wide significance threshold of 4.84 × 10^−6^ (0.05/10329) and a suggestive linkage threshold of 9.68 × 10^−5^ (1/10329). Manhattan plots of the GWAS results for each trait were produced with the qqman package available from R (Version 3.1.2) [19].

### QTL mapping

Single QTL detection analyses were carried out with the QTLMap software [20], which was developed to handle data on F2 individuals for designs from outbred populations. QTLMap is an interval mapping method, in which QTL are detected using a likelihood ratio test (LRT) calculated under the hypothesis of one versus no QTL linked to the given SNPs. The linkage disequilibrium linkage analysis (LDLA) option of QTLMap was applied to single trait [17]. No assumptions were made on the fixation of alleles in the grand-parental lines (L2 and R-) and on the number of segregating alleles at the QTL. The family structure of the F2 population consisted of six half-sib families, and the minimum size for considering dam families was set at 20 progeny, which was the case for 15 of the 51 dam families. Fixed effects of dam, sex, batch, as well as covariates (BW00 or BW08 depending on the trait studied) were taken into account in the model. For the Z chromosome, an interaction between the QTL and the fixed effect of sex was added to the model. The LRT value was calculated by scanning chromosomes for QTL with a step of 1 cM. The method proposed by Churchill and Doerge [23] was used to estimate empirical thresholds taking into account the family structure (pedigree) and genotype data (SNPs). Chromosome-wide significance of LRT values was obtained by 10,000 simulations under the null hypothesis (no QTL) to calculate 1 and 5% significance thresholds [21]. Genome-wide thresholds were defined by applying the Bonferroni correction across chromosomes:

$${\text{P}}_{\text{genome} -\text{wide}} = 1 - \left( {1 - {\text{P}}_{\text{chromosome} -\text{wide}} } \right)^{n},$$

where *n* is the number of chromosomes used in the study. Confidence intervals for QTL were estimated by the one LOD drop-off method [22]. The QTL substitution effect was assessed in each sire family at the position of the LRT maximum, and significance was assessed by Student’s *t* test. The additive value of a QTL effect was calculated as the mean of the absolute value of the sire substitution effects (*P* value < 0.05). SNP positions and information were annotated using the GalGal4 genome assembly [24]. Information on potential candidate genes in each QTL region was searched for in the NCBI and Ensembl databases [24, 25]. Furthermore, for genes that were associated with significant SNPs, we performed a search of the gene ontology database [26] to draw hypotheses about the biological processes and molecular functions that likely influence the trait of interest.

### Bioinformatics

In previous research, whole-genome sequence data was produced for one L2 chicken [27] and for two R- chickens [28]. The sequenced animals were chosen at random in a recent generation for each line and constitute a resource for identifying SNPs that differ between the lines but were not used as F0 animals in the current F2 design.

Sequence alignment between the reference genome assembly GalGal4 and the whole-genome sequences of the L2 and R- individuals was performed to identify SNPs that differ between the two lines. Sequencing fragments with a Phred quality score lower than 15 and the adapter sequences were removed by Trimmomatic [29] before alignment to the chicken reference genome assembly Galgal4.84 [25]. Trimmed reads that were shorter than 36 bp were then eliminated. Only read pairs for which both forward and reverse reads remained after trimming were mapped to the chicken reference by Burrows–Wheeler Alignment tool (BWA) [30].

### SNP calling

Genome Analysis Toolkit (GATK) was applied to identify variants across the genome [31–33]. Mutations that were located within repeated sequences were marked by Picard [34] and discarded. In order to increase the accuracy of insertion and deletion (InDel) calling, reads around InDels were locally realigned by the IndelRealigner tool provided by GATK. The HaplotypeCaller tool of GATK was applied to detect variants. A read base with a Phred score less than 10 was not considered in the variant discovery step. Only mutations that were called with a Phred score equal to or higher than 30 were considered as confident mutations and reported. Our search for SNPs that differed between the L2 and R- birds was focused on the QTL regions that were detected by both GWAS and QTL mapping, with the aim to identify potential candidate mutations for further study. Functional effects of the identified mutations were predicted by SnpEff [35] and with Variant Effect Predictor (VEP) [36].

## Results

### Phenotypic means of the F0 and F2 populations

None of the phenotypes recorded in this study significantly deviated from the normal distribution (*P* > 0.05). Means and standard deviations for the F0 lines and their F2 crosses are in Tables 1 and 2 for males and females, respectively. Highly significant differences were found between the L2 and R- lines for all growth traits (BW00 to BW16) and for CA16. Lines did not differ for immune response to sheep red blood cells, except for SRBC14 which was lower in R- females (*P* < 0.05) than in L2 females. Shank length was not available for the F0 individuals. Animals from the R- line were lighter than those from the L2 line, and also had a much smaller comb area. In the F2 generation, the fixed effects of sex, dam, and batch were significant, except for sex for BW00 and SRBC00, batch for BW08, and dam for BW1612, SRBC07, and SRBC14. The R- square values for the model ranged from 0.11 (SRBC00) to 0.77 (CA16). Regression coefficients on BW00 or BW08 were not significant for any trait (*P* > 0.10). Coefficients of variation for body weight traits and shank length ranged from 10 to 20% but were much higher for comb area (about 40 to 50% depending on sex) and for immune response traits (about 50 to 70%).

**Table 1:** Body weight (g), comb area (cm^2^), shank length (cm), and immune response traits (SRBC) in the F0 L2 and R- lines and their F2 male progeny from hatch to 16 weeks of age

Male	L2		R-		*p* value	F2	
Trait	Mean	SD	Mean	SD		Mean	SD
BW00	27.5	2.1	36.3	1.5	<0.0001	32.2	2.3
BW04	354.8	46.4	238.7	26.4	<0.0001	290.7	51.3
BW08	1052.5	113.2	759.7	75.3	<0.0001	937.0	131.3
BW12	1758.4	240.3	1309.4	106.0	<0.0001	1511.6	262.4
BW16	2112.4	196.1	1808.1	128.4	0.0008	2220.4	324.7
CA16	56.6	12.1	16.0	2.4	<0.0001	31.8	13.1
SL08	–	–	–	–	–	7.0	0.5
SRBC00	–	–	–	–	–	0.3	0.8
SRBC07	5.2	2.1	4.9	1.6	0.79	2.6	1.8
SRBC14	4.1	1.8	3.3	1.2	0.97	2.7	1.7

BW00, 04, 08, 12, 16: body weight at hatch, at 4, 8, 12, and 16 weeks of age; CA16: size of comb area at 16 weeks of age; SRBC00, 07, 14: baseline antibody level, antibody level at 7 and 14 days after primary immunization of sheep red blood cell; SD: standard deviation; the *p* value corresponds to the significance level of the comparison between L2 and R- lines, for each trait

**Table 2:** Body weight (g), comb area (cm^2^), shank length (cm), and immune trait (SRBC) in the F0 L2 and R- lines and their F2 female progeny from hatch to 16 weeks of age

Female	L2		R-		*p* value	F2	
Trait	Mean	SD	Mean	SD		Mean	SD
BW00	25.7	2.5	35.8	1.9	<0.0001	32.4	2.5
BW04	285.1	40.2	220	28.7	0.0014	256.5	48.3
BW08	829.2	86.1	610	96.0	<0.0001	735.2	148.1
BW12	1312.4	126.7	933.1	128.6	<0.0001	1147.1	249.2
BW16	1675.5	143.7	1170.4	195.6	<0.0001	1526.5	309.2
CA16	10.2	5.3	3.0	1.0	0.0022	4.8	2.5
SL08	–	–	–	–	–	6.2	0.7
SRBC00	–	–	–	–	–	0.3	0.8
SRBC07	5.6	1.5	5.4	1.3	0.78	3.1	2.2
SRBC14	5.0	1.4	3.4	1.0	0.01	3.1	1.7

BW00, 04, 08, 12, 16: body weight at hatch, at 4, 8, 12, and 16 weeks of age; CA16: size of comb area at 16 weeks of age; SRBC00, 07, 14: baseline antibody level, antibody level at 7 and 14 days after primary immunization of sheep red blood cell; SD: standard deviation; the p-value corresponds to the significance level of the comparison between L2 and R- lines, for each trait

### Population stratification

The *F*_st_ between the parental lines was equal to 0.31, which shows significant differentiation between the lines. The structure of the F2 population was assessed by MDS analysis by applying an r^2^ less than 0.2. Plotting of the first two MDS components showed that F2 individuals clustered according to mating type (LR or RL F1 males) and half-sib family (four families from the LR males and two families from the RL males), which showed that the MDS components represented the family structure of the F2 generation (see Additional file 5: Figure S4). As expected, clustering of individuals within sires reflected the within-sire dam family structure (see Additional file 6: Figure S5).

### Genome-wide association analysis

**Fig. 1:**
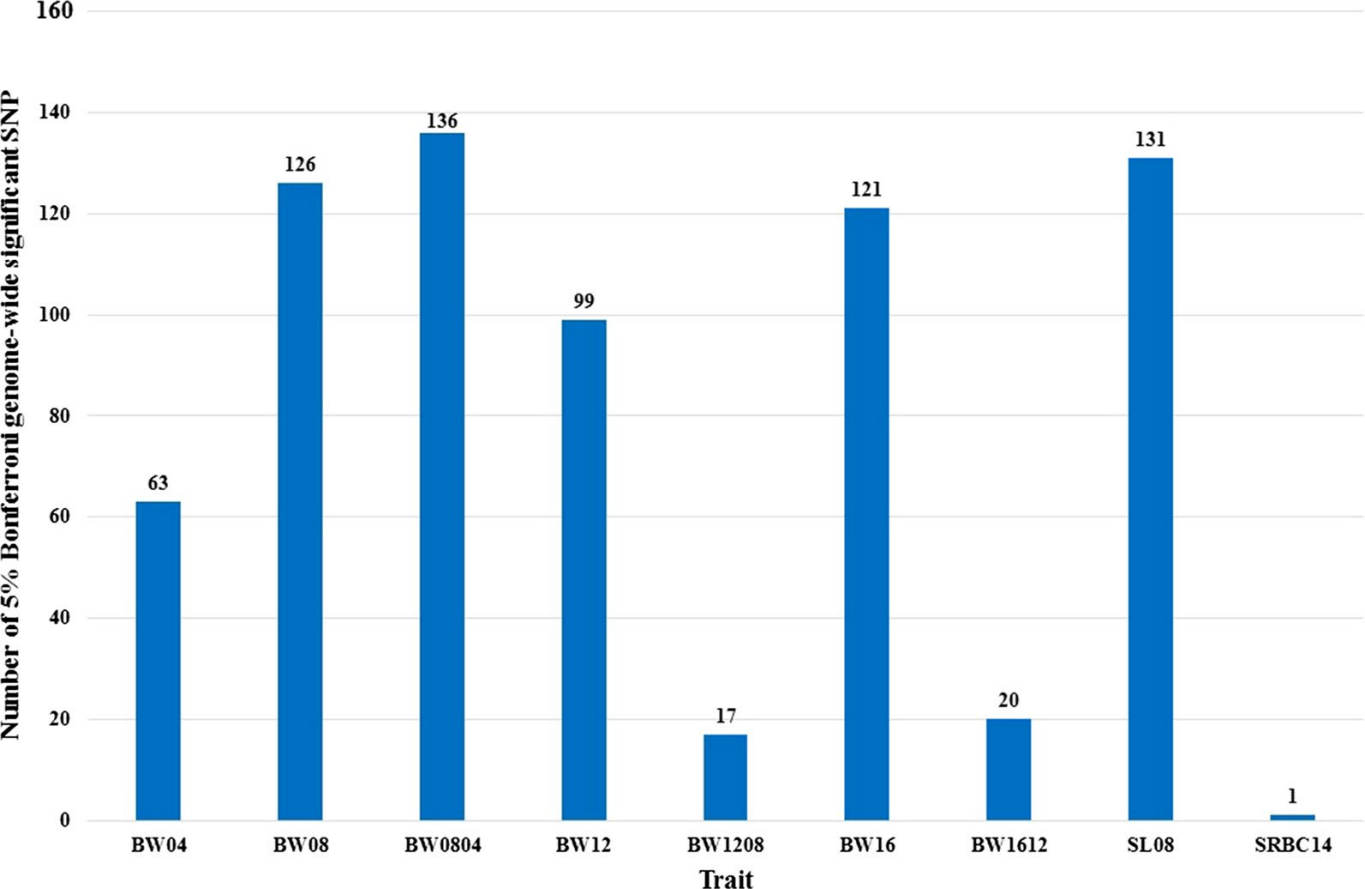
Number of SNPs reaching 5% Bonferroni genome-wide significance (*P* value <4.84 × 10^−6^) for the nine evaluated traits. A total of 714 effects associated with 178 SNPs were identified for nine traits at the 5% Bonferroni genome-wide significance level (*P* value <4.84 × 10^−6^). No SNP effect reached 5% Bonferroni genome-wide significance for BW00, CA16, SRBC00, and SRBC07. BW0804 had the largest number of SNP effects (136), followed by SL08 (131) and BW08 (126)

A total of 714 SNP effects associated with 178 SNPs were identified for nine traits with 5% Bonferroni genome-wide significance (*P* value <4.84 × 10^−6^) (see Additional file 7: Table S2). No SNP effect reached 5% Bonferroni genome-wide significance for BW00, CA16, SRBC00, and SRBC07. BW0804 had the largest number of genome-wide significant SNP effects (136), followed by SL08 (131) and BW08 (126) (Fig. 1). In contrast, a limited number of significant SNP effects was detected for SRBC14 (1), BW1208 (17), and BW1612 (20). In addition, 364 SNP effects associated with 234 SNPs reached suggestive significance (*P* value <9.68 × 10^−5^).

**Fig. 2:**
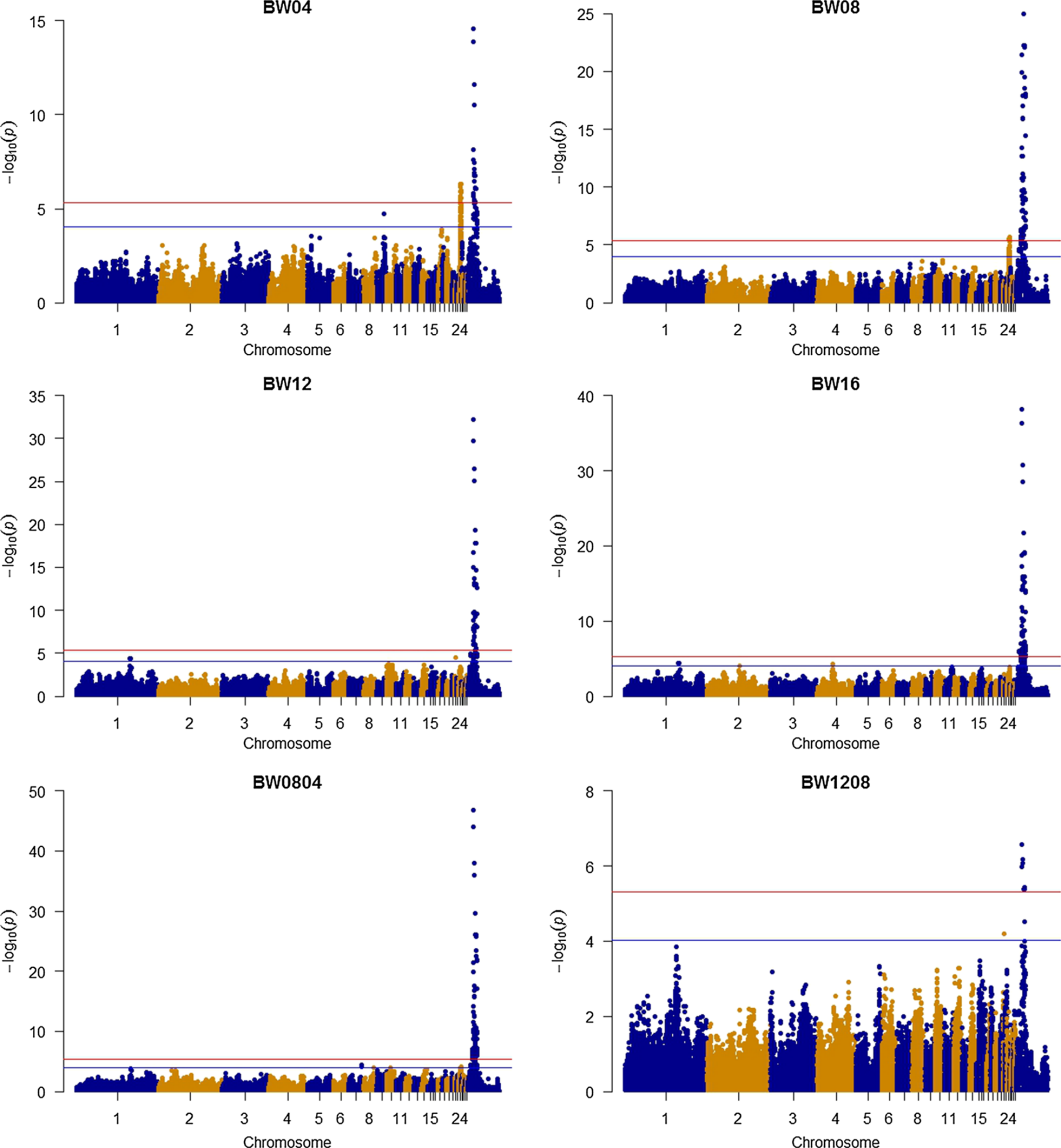
Manhattan plots. Manhattan plots for genome-wide analyses of BW04, BW08, BW12, BW16, BW0804, and BW1208. *Red* and *blue lines* correspond to thresholds of 4.84 × 10^−6^ and 9.68 × 10^−5^, respectively

**Fig. 3:**
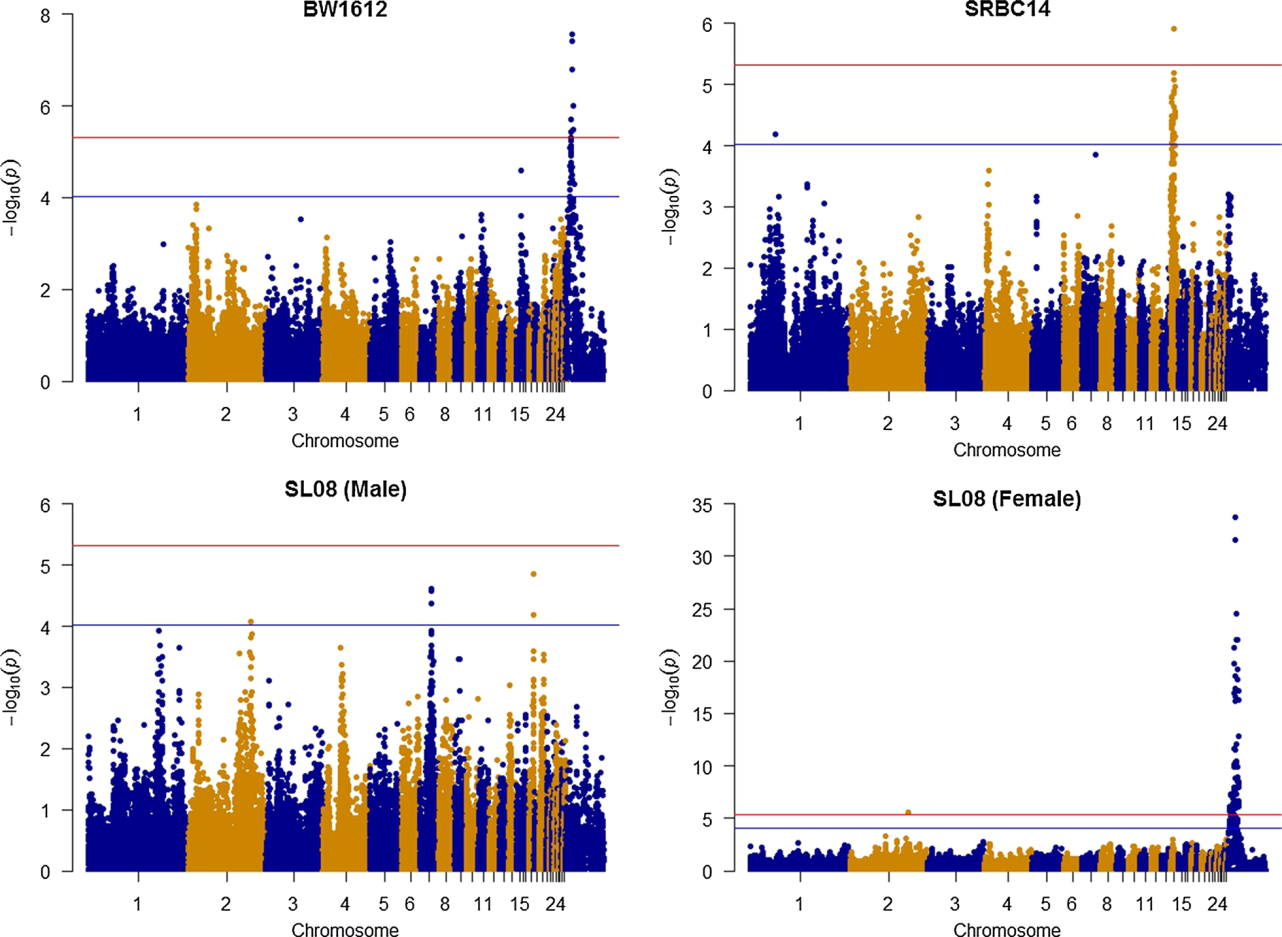
Manhattan plots. Manhattan plots for genome-wide analyses of BW1612, and SL08. *Red* and *blue lines* correspond to thresholds of 4.84 × 10^−6^ and 9.68 × 10^−5^, respectively

Only three chromosomes (GGA14, 24, and Z) carried genome-wide significant SNPs (Figs. 2, 3), whereas suggestive SNP effects were found on 11 chromosomes (GGA1, 2, 4, 7, 9, 14, 15, 22, 24, 26, and Z) (Table 3). GGAZ carried the most genome-wide significant SNP effects, which were detected only in females and for eight traits (BW04, BW08, BW12, BW16, BW0804, BW1208, BW1612, and SL08). The strongest association was found for a large region on GGAZ that spanned 13.1 Mb (between 8.9 and 22.0 Mb) and contained 146 genome-wide significant SNPs associated with eight growth-related traits. The most significant SNPs (3.57 × 10^−42^ < *P* value <1.74 × 10^−47^) were associated with BW08, BW0804, and SL08. This region contained 30 SNPs, harbored three annotated genes and one uncharacterized gene. Among these, the highest significance (*P* value ≤1.74 × 10^−47^) was obtained for SNPs in the *prostaglandin E receptor 4* (*PTGER4*) and *complement component 7* (*C7*) genes and in the uncharacterized gene (*LOC100857889*). Two overlapping chromosomal regions on GGA24 (between 4.1 and 5.3 Mb and between 4.3 and 6.0 Mb) were associated with BW04 and BW08, respectively. These two regions harbored 13 and seven genome-wide significant SNPs, respectively, and have been reported to carry several growth-related QTL [37]. For SRBC14, we detected one genome-wide significant SNP (rs10724420) that was flanked by suggestive SNPs. This SNP was located in the 3′-UTR of the *3*-*phosphoinositide dependent protein kinase 1* gene (*PDPK1*), which is involved in transcriptional regulation of proopiomelanocortin in mice, with consequences for food intake, body weight and size [38]. This region on GGA14 was previously reported in studies on body weight and immune related QTL [37].

**Table 3:** List of QTL and genome-wide significant SNPs identified by QTL mapping (LDLA) and GWAS

Trait	GGA	Loc (M)	CI (M)	MLR	Nearest SNPs	SL	QTL effect	GWAS	Published related QTL
BW00	1	3.452	3.442–3.462	124.22	GGaluGA046916-rs13947322	G***	0.269	–	BW
BW00	2	0.831	0.821–0.841	74.46	rs14168592-rs14170121	G*	0.251	–	BW
BW04	2	2.341	2.331–2.351	60.68	rs14234623-rs16106127	C*	0.498	–	BW
BW08	2	2.341	2.331–2.351	71.7	rs14234623-rs16106127	C**	0.748	–	BW
BW16	2	2.341	2.331–2.351	82.98	rs14234623-rs16106127	G**	0.720		BW
SL08	2	2.341	2.331–2.351	63.31	rs14234623-rs16106127	C*	0.486	–	BW
SRBC00	2	0.111	0.101–0.141	60.69	GGaluGA131350-rs14134125	C*	0.431	–	BW
BW12	3	0.06	0.05–0.07	65.9	rs14308283-GGaluGA203599	C**	0.399	–	BW
BW16	3	0.03	0.02–0.07	62.52	GGaluGA203198-GGaluGA203599	C*	0.345	–	BW
CA16	3	0.02	0.01–0.03	66.44	rs14309145-rs14308726	C**	0.280	–	BW
BW00	4	1.141	1.131–1.151	77.01	GGaluGA258962-rs14469696	G*	0.787	–	BW,IM
SRBC14	4	1.391	1.381–1.401	63.77	rs14480306-GGaluGA262668	C**	0.622	–	BW
CA16	5	0.231	0.221–0.241	60.79	rs14511113-rs15655840	C**	0.426	–	IM
SL08	5	0.611	0.601–0.621	54.24	GGaluGA278486-rs14521985	C*	0.343	–	BW
BW04	7	0.732	0.722–0.742	60.69	rs16596997-rs15864433	C**	0.514	–	BW
SL08	7	0.712	0.702–0.732	58.74	GGaluGA315581-GGaluGA316229	C*	0.221	–	BW
BW00	9	0.774	0.764–0.784	139.65	GGaluGA342923-rs16677966	G***	0.449	–	BW,IM
SL08	12	0.103	0.093–0.113	57.16	rs13621525-GGaluGA082232	C*	0.469	–	BW
SRBC00	12	0.403	0.393–0.413	55.25	rs14040595-GGaluGA085745	C*	0.385	–	BW
SL08	13	0.077	0.067–0.087	52.39	rs14057266-GGaluGA091278	C*	0.421	–	BW
SRBC00	13	0.317	0.307–0.327	54.13	GGaluGA094109-rs15001109	C*	0.496	–	BW
BW04	14	0.131	0.121–0.141	108.62	rs15722248-rs15723606	G***	0.526	–	BW,IM
BW08	14	0.171	0.151–0.181	69.68	rs14071741-GGaluGA100621	C**	0.453	–	BW
SL08	14	0.171	0.161–0.181	64.13	rs13531412-GGaluGA100621	C*	0.188	–	BW
SRBC14	14	–	–	–	–	–	–	1 G-W SNP (7418129 bp)	–
BW16	17	0.277	0.257–0.287	51.51	rs15799059-rs14100782	C*	0.567	–	BW
CA16	17	0.137	0.127–0.147	50.45	rs15033487-GGaluGA113549	C*	0.310	–	BW
SRBC00	17	0.197	0.187–0.207	62.72	rs15032558-rs14102021	C**	0.363	**–**	BW
SRBC07	18	0.412	0.402–0.422	49.89	rs13509086-GGaluGA122291	C*	0.177	**–**	–
SRBC14	18	0.412	0.402–0.422	50.99	rs13509086-GGaluGA122291	C*	0.442	**–**	–
SRBC14	19	0.151	0.141–0.161	56.17	rs13573938-rs14118340	C**	0.349	**–**	BW
BW12	20	0.381	0.371–0.391	52.76	rs16005422-rs16172318	C*	0.446	–	BW
SL08	20	0.381	0.371–0.391	51.38	rs16005422-rs16172318	C*	0.233	–	BW
SRBC00	21	0.3	0.29–0.33	55.45	rs16180505-GGaluGA184295	C*	0.289	**–**	–
BW04	24	0.291	0.281–0.301	53.35	rs16196993-GGaluGA192626	C*	0.214	13 G-W SNPs (4145811–5321244 bp)	BW
BW08	24	–	–	–	–	–	–	7 G-W SNPs (4286369–6019223 bp)	–
CA16	24	0.281	0.271–0.291	54.07	GGaluGA192284-rs14295712	C*	0.325	–	BW
CA16	26	0.385	0.375–0.395	52.51	rs14300692-rs16204214	C*	0.182	–	BW
BW00	27	0.461	0.451–0.471	117.61	GGaluGA200219-rs14304325	G***	0.402	–	BW,IM
BW00	28	0.203	0.193–0.213	49.59	GGaluGA201613-GGaluGA201725	C*	0.556	–	BW,IM
BW04	Z	0.43	0.41–0.44	166.33	rs14756962-rs16763234	G***	0.893	50 G-W SNPs (11113817–17739555 bp)	IM
BW08	Z	0.38	0.36–0.39	294.24	rs14786654-rs16102663	G***	1.409	119 G-W SNPs (9581584–21954151 bp)	BW,IM
BW0804	Z	0.38	0.36–0.39	288.44	rs14786654-rs16102663	G***	1.323	136 G-W SNPs (9088090–21954151 bp)	BW,IM
BW12	Z	0.38	0.36–0.39	226.51	rs14786654-rs16102663	G***	1.712	99 G-W SNPs (10983124–21146543 bp)	BW,IM
BW1208	Z	–	–	–	–	–	–	17 G-W SNPs (11327074–17739555 bp)	–
BW16	Z	0.37	0.35–0.39	216.29	rs16131361-rs16102663	G***	1.773	121 G-W SNPs (4646914–21954151 bp)	BW,IM
BW1612	Z	0.32	0.31–0.33	75.71	GGaluGA347787-rs16780992	C*	1.158	20 G-W SNPs (8872779–15196080 bp)	–
CA16	Z	1.1	1.09–1.13	82.4	rs16109845-rs16768163	C*	0.615	–	BW,IM
SL08	Z	0.37	0.36–0.39	367.79	rs14786654-rs16102663	G***	2.250	131 G-W SNPs (2937208–115665705 bp)	BW,IM
SRBC07	Z	0.6	0.59–0.61	79.81	rs16100247-GGaluGA349037	C*	0.756	–	IM

GGA: *Gallus gallus* chromosome; Loc: location; CI: confidence interval; MLR: maximal likelihood ratio; SL: significance level with C*, C**, and C*** at 5, 1, and 0.1% chromosome-wide significance and G*, G**, and G*** at 5, 1, and 0.1% Bonferroni genome-wide (G-W) significance, respectively; QTL effect: substitution effect expressed in residual standard deviation; GWAS: chromosomal region detected by GEMMA; BW00, 04, 08, 12, 16: body weight at hatch, at 4, 8, 12, and 16 weeks of age; BW0804, 1612: body weight gain during 4 to 8 weeks of age and 12 to 16 weeks of age; CA16: size of comb area at 16 weeks of age; SL08: shank length at 8 weeks of age; SRBC00, 07, 14: baseline antibody level, antibody level at 7 and 14 days after primary immunization of sheep red blood cell; Published related QTL: QTL regions that were detected in the current study and overlapped with published related QTL (BW: overlapped with body weight, shank length, or egg weight QTL; IM: overlapped with immune related QTL

### QTL mapping

The whole-genome single QTL analysis led to the identification of 47 QTL that corresponded to 34 non-overlapping regions on 20 chromosomes (Table 3), since there were several overlapping QTL regions for different traits. Thirteen of these 47 QTL had high genome-wide significance (*P* < 0.05) and corresponded to 10 non-overlapping regions on GGA1, 2, 4, 9, 14, 27, and Z. BW00 was associated with the largest number of genome-wide QTL regions, which were distributed on five chromosomes (GGA1, 2, 4, 9, and 27), whereas the QTL regions for other traits were distributed on GGA2 (BW12), GGA14 (BW04), and GGAZ (BW04, BW08, BW12, BW16, BW0804, and SL08). The QTL that were the most highly significant at the chromosome-wide level (*P* value <0.01) affected BW04 (GGA7), BW08 (GGA2 and GGA14), BW12 (GGA3), CA16 (GGA3 and 5), SRBC00 (GGA17), and SRBC14 (GGA4 and 19). All other identified QTL had chromosome-wide significance *P* values less than 0.05. Estimates of QTL allele substitution effects ranged from 0.18 (SRBC07 on GGA18) to 2.25 (CA16 on GGA26) residual standard deviations, with a mean of 0.58 standard deviations. Among the autosomes, GGA2 carried the largest number of detected regions, with six identified QTL, followed by GGA3, 4, and 17, which each carried three QTL. The average confidence interval spanned 2.4 cM, ranging from 2 to 5 cM.

Three QTL with chromosome-wide significance that affected SRBC traits on GGA18 and 21 were not reported before. The chromosome-wide QTL found for CA16 on GGA3 was not located at the same position as the one previously described on that chromosome for comb mass [4].

### QTL detected by both GWAS and QTL mapping-LDLA

The position of the SNPs that flanked a QTL region was used to establish the correspondence between the results of QTLMap and those of GEMMA, by considering all SNP effects detected at a *P* value less than 0.05. Regions detected by both methods were found only on two chromosomes: GGA24 for BW04 and GGAZ for seven traits (BW04, BW08, BW12, BW16, BW0804, BW1612, and SL08). Thus, of the 47 QTL detected with QTLMap, eight were also detected with GEMMA. The 39 QTL that were detected only with QTLMap corresponded to 35 chromosome regions that overlapped with previously published QTL, for either growth- or immune-related traits [37].

### Sequence analysis

The analysis of whole-genome sequence data focused on the 13 genome-wide significant QTL (10 non-overlapping chromosomal regions) detected by QTLMap, and the region identified with GEMMA on GGA24, which contained genome-wide significant SNPs but was only chromosome-wide significant with QTLMap. The L2 bird and the two R- birds differed for 15,443 SNPs in 106 genes (83 known genes, 16 genes coding for uncharacterized proteins, and seven miRNA); 1698 of these SNPs were novel. Among these 106 genes, 21 carried 41 missense mutations, as identified by VEP. The results are summarized according to QTL region in Table S3 (see Additional file 8: Table S3). An in-depth analysis focused on genes with known functions that were the closest to the position showing the highest probability in QTL mapping or GWAS.

Two genome-wide significant QTL were detected on GGA2 for body weight traits. The QTL that affected BW00 (between 41.1 and 42.4 Mb) contained 18 genes, of which four *abhydrolase domain containing 5* (*ABHD5*), *calpain 7* **(** *CAPN7*), *maturin, neural progenitor differentiation regulator homolog* (*MTURN*), and *testis and ovary specific PAZ domain containing 1* (*TOPAZ1*), are involved in the regulation of body weight, total amount of body fat, and lean body mass in mice [39]. The *TOPAZ1* gene carried 207 SNPs that differed between the L2 and the R- birds, of which 80 were novel SNPs, and one missense SNP that resulted in a valine being replaced by an isoleucine at position 431 of the protein. The QTL that affected BW16 (between 111.2 and 112.0 Mb) harbored two genes, *cytochrome P450, family 7, subfamily a, polypeptide 1* (*CYP7A1*) and *inositol monophosphatase domain containing 1* (*IMPAD1*), which are associated with birth weight [40] and increased body weight [39] in mice.

The region on GGA14 (between 2.8 and 3.3 Mb) was associated with a strong effect for BW04 and carried the *ACTB* gene, which is involved in the regulation of body weight and size in mice [41]. This gene contained 75 SNPs that differed between the L2 and the R- birds, of which 12 were novel SNPs. Three QTL on GGAZ that contain several growth-related genes were detected in this study for BW16 (between 11.7 and 13.0 Mb), BW04 (between 13.7 and 14.7 Mb), and BW08, BW0804, BW12, SL08 (between 12.1 and 13.0 Mb). The *PTGER4* gene carried 104 SNPs that differed between the L2 and the R- birds, of which 96 were novel SNPs. A previously described missense mutation (rs315266117, 12708649 bp) was detected in the genome of the R- birds; it is located close to the SNP with the lowest P-value in our GWAS analysis and results in the replacement of a glutamic acid by a lysine at position 375 of the protein, which is deleterious according to the SIFT prediction. The *OSMR* gene, which is involved in muscle organ and skeletal development in chickens [36], carried 144 SNPs that differed between the L2 and the R- birds, of which 136 were novel SNPs. One missense mutation (rs312389473) that resulted in the replacement of a threonine by a serine was located at position 501 of the protein. Among the other identified genes, *PRKAA1* (*protein kinase, AMP*-*activated, alpha 1 catalytic subunit*) and *RICTOR* (*RPTOR independent companion of MTOR, complex 2*) are related to viscera weight [42, 43] and *prostaglandin E receptor 4* (*EP4*), *fibroblast growth factor 10* (*FGF10*), and *leukemia inhibitory factor receptor* (*LIFR*), which are located in the QTL region for BW16 (between 11.7 and 13.0 Mb on GGAZ), have been reported to be associated with body weight related traits [44, 45]. *FGF10* and *EP4* contained 189 and 106 SNPs that differed between the L2 and R- lines and each of these genes carried one missense SNP.

Furthermore, three QTL for BW00, on GGA4, 9, and 27, harbored four growth-related genes, among which *ubiquitin*-*like modifier activating enzyme 6* (*UBA6*) on GGA4 and *MDS1 and EVI1 complex locus* (*MECOM*) on GGA9 play a critical role in body size/length and weight loss in mice [46, 47]. The QTL region on GGA27 contained two genes that regulate decrease in body weight in mice, i.e., *LIM and SH3 protein 1* (*LASP1*) and *phosphatidylinositol*-*5*-*phosphate 4*-*kinase, type II, beta* (*PIP4K2B*) [48, 49].

## Discussion

In this study, we focused on traits that were measured during the growing period and are likely to play an important role in the adaptation of chickens to sub-optimal conditions, particularly to tropical climate. Since the lines used for the F2 design were original experimental lines, our findings may be specific to these genetic backgrounds and/or to the sub-optimal environmental conditions that differ from other studies. Yet, most of the QTL regions detected for growth traits overlapped with previously published QTL, which suggests that these QTL have an effect across a range of environmental conditions and could be particularly useful for selection. Several genome-wide QTL were detected for BW00, a trait that is strongly influenced by maternal effects. Since data on egg weight of the F1 dams were not available at the time of reproduction, this effect could not be separated from the additive dam effect on chick weight.

Surprisingly, no genome-wide significant QTL effects were detected on comb area, a trait that is likely relevant for climate adaptation and that differed significantly between the parental lines. Because of the large sexual dimorphism of this trait, we analyzed the data from F2 males and females separately to search for sex-specific QTL but none were detected. This may be explained by measurement errors, which can be quite important for both sexes, or by the rather large number of QTL that control variation of this trait, each with a small effect. None of the regions previously described for comb mass [4] were identified in the current study, which could be due to the use of lines with different genetic backgrounds in the current study.

Very little overlap was observed between the QTL regions detected by QTLMap and GEMMA. The differences between GWAS and QTL mapping-LDLA may be due to GWAS detecting associations based on LD across the F2 population, whereas QTL mapping-LDLA also exploits the information provided by within-family segregation, in order to estimate SNP effects on the trait variation. Legarra et al. [50] compared LDLA and EMMA methods (such as GEMMA) using real and simulated datasets and showed good agreement for the location of QTL between them. In general, these datasets consisted in a large number of small families, except for the sheep dataset, in which five F1 sires were mated to a large number of F1 dams, so that the number of half-sib families was similar to the design in the current study but the proportion of full-sibs was much lower in the sheep dataset. In our case, differences between the GWAS and LDLA results could be due to the fact that within-family segregation provides the most relevant information for QTL detection.

Although GEMMA and QTLMap identified the same QTL region on GGAZ, with strong effects on body weight and shank length, it was significant only in females with GEMMA, while QTLMap offered the possibility to include an interaction between sex and QTL. Such detection of sex-specific QTL is not new and they could contribute to our results, but in the present case, this result could also be due to the segregation mode of Z-linked alleles according to sex. In females, only two alleles are compared since females are hemizygous, so this is a 1:1 comparison of an R- allele to an L2 allele, whereas in males the within-family comparison will differ according to mating type. When F1 females have inherited their Z chromosome from an L2 F0 female (named Z_L2_), the F2 male progeny can have two possible genotypes: homozygous Z_L2_Z_L2_ or heterozygous Z_L2_Z_R-_, with some recombination occurring on the Z chromosome inherited from the F1 male. When F1 females inherit their Z chromosome from an R- F0 female (named Z_R-_), the F2 male progeny scan have two possible genotypes: homozygous Z_R–_Z_R-_ or heterozygous Z_L2_Z_R-_, with recombination occurring on the Z chromosome inherited from the F1 male. Thus, if the QTL effect is not additive, the different F2 families will not provide the same contribution to estimation of the QTL, which may result in the absence of a significant association. Thus, GWAS involving sex-linked inheritance may require specific analysis.

Relevant candidate genes were identified in this study, according to their position and function. Most of them belong to networks of genes that are involved in embryonic, organism and tissue development, according to Ingenuity Pathway Analysis (IPA) [51]. The sequence data available from previous studies provided a list of SNPs that represents only a subset of all SNPs that can differ between the lines. Some of the alleles at these SNPs are expected to be line-specific as a result of the long selection history of each line and the genetic distance between the lines. Since line-specific alleles may arise because of selection response or because of random drift, follow-up studies are necessary to validate whether or not these SNPs contribute to the QTL effect. Since missense mutations are rare, they could be the first choice for validation analyses, but it is also expected than non-coding variants can be quite relevant for quantitative variation. Thus, the choice of SNPs to be validated will be made according to their position on the gene, heterozygosity in the F1 population, and technical factors depending on the genotyping method.

For instance, *PTGER4*, a candidate gene for the growth QTL on GGAZ, has no known function in chicken but plays an important role in osteoclast differentiation and physiology in mice [52–54]. Although the role of this gene is not well known in chickens, it is a good candidate for further analysis, starting with the genotyping of F1 and F2 animals for SNPs chosen from the sequence data, followed by expression studies in each parental line if a significant effect on growth is identified in the F2 animals. Furthermore, genotyping candidate SNPs in the F2 population would also help to understand the different results obtained for F2 males and females. An obvious candidate gene to be tested for the QTL on GGAZ is the *growth hormone receptor* gene, which is known to carry mutations that cause sex-linked dwarfism in chickens. This gene lies close to the QTL region but not in it, so it may be worthwhile to include it in a confirmation study.

Our findings can also be useful for the future management of the L2 and R- lines. The frequency and the phenotypic consequences of the validated SNPs should be determined in the current breeding populations of the L2 and R- lines, to investigate their potential use in the management of these lines. Depending on their frequency and effect, some of these SNPs could be useful in the selection process for sub-tropical climate adaptation. Another option would be to set up a dual-purpose cross, taking advantage of the sex-linked QTL on growth. For instance, R- males could be crossed to L2 females to take advantage of heterosis in hybrids by producing light weight F1 females for egg production and F1 males with a greater body weight for local broiler production.

## Conclusions

We have identified QTL for growth and morphological traits that may influence adaptation of chickens to varying environmental conditions. The availability of whole-genome sequence data for each parental line was useful to better document the candidate genes that were identified according to their positions and known functions. Finally, the very strong QTL effects found on the Z chromosome for body weight and shank length underlines the importance of sex-linked inheritance for growth-related traits in chickens, which is particularly relevant for crossbreeding in poultry breeding programs.

## Additional files

**Additional file 1: Figure S1.** Distribution of dam family sizes.

**Additional file 2: Table S1.** Calendar of production of the F2 generation.

**Additional file 3: Figure S2.** Variation in ambient temperature in the surrounding area of the experimental farm at the time of the experiment.

**Additional file 4: Figure S3.** Calendar of vaccination plan and immune experiment.

**Additional file 5: Figure** **S4.** Clustering of F2 individuals according to F1 sire families (6 sires).

**Additional file 6: Figure** **S5.** Clustering of F2 individuals according to F1 dam families (32 dams).

**Additional file 7: Table S2.** Results of the GWAS: list of SNPs showing 5% Bonferroni genome-wide significance for the recorded traits.

**Additional file 8: Table S3.** Results of the whole-genome sequence analysis: list of genes showing SNPs between the L2 and the R- birds in the genome-wide QTL regions detected by either QTLMap or GEMMA. SNPs were classified according to their position in the gene.
