# Supplementary figures and images for: Detection of QTL for traits related to adaptation to sub-optimal climatic conditions in chickens
Source: Genet Sel Evol. 2017 Apr 20;49:39. doi: 10.1186/s12711-017-0314-5 (PMC5399330; doi:10.1186/s12711-017-0314-5)

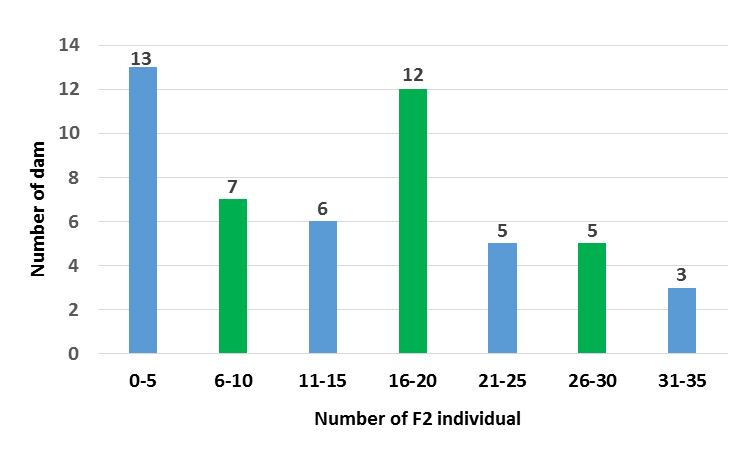


Fig. S1 The distribution of dam family size

Supplement: Supplementary file 1 — Additional file 1: Figure S1. Distribution of dam family sizes. [file 12711_2017_314_MOESM1_ESM.docx]

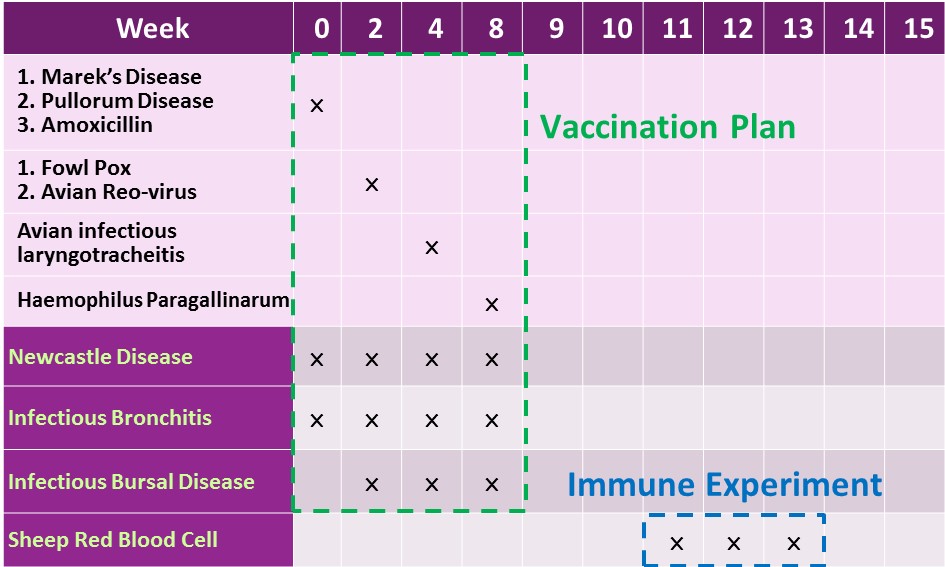


Fig. S3 The calendar of vaccination plan and immune experiment

Supplement: Supplementary file 4 — Additional file 4: Figure S3. Calendar of vaccination plan and immune experiment. [file 12711_2017_314_MOESM4_ESM.docx]

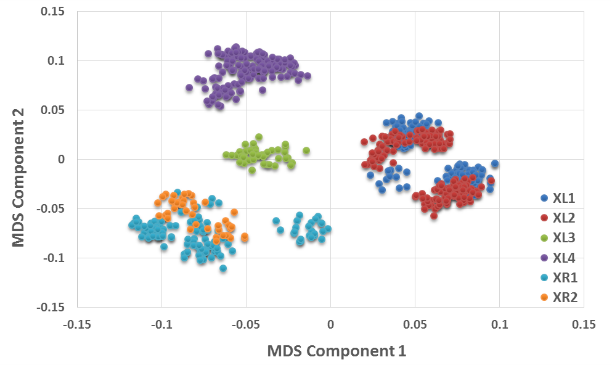


Fig. S4 Clustering of F2 individuals according to F1 sire families (6 sires)

Supplement: Supplementary file 5 — Additional file 5: Figure S4. Clustering of F2 individuals according to F1 sire families (6 sires). [file 12711_2017_314_MOESM5_ESM.docx]

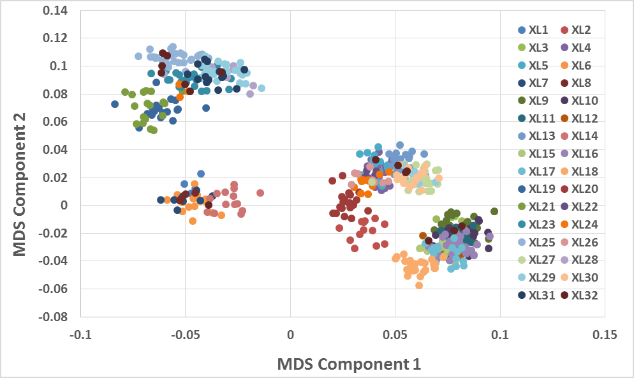


Fig. S5 Clustering of F2 individuals according to F1 dam families (32 dams)

Supplement: Supplementary file 6 — Additional file 6: Figure S5. Clustering of F2 individuals according to F1 dam families (32 dams). [file 12711_2017_314_MOESM6_ESM.docx]
