# Supplementary material for: Detection of QTL for traits related to adaptation to sub-optimal climatic conditions in chickens
Source: Genet Sel Evol. 2017 Apr 20;49:39. doi: 10.1186/s12711-017-0314-5 (PMC5399330; doi:10.1186/s12711-017-0314-5)
Supplement: Supplementary file 2 — Additional file 2: Table S1. Calendar of production of the F2 generation. [file 12711_2017_314_MOESM2_ESM.docx]

Table S1 The calendar of production of the F2 generation

| **Batch** | **Birth date** | **Date of 16 weeks of age** | **Climatic condition in Taiwan** |
| --- | --- | --- | --- |
| 1 | 17.12.2010 | 08.04.2011 | Winter-Spring |
| 2 | 31.12.2010 | 22.04.2011 | Winter-Spring |
| 3 | 18.01.2011 | 10.05.2011 | Winter-Spring |
| 4 | 18.02.2011 | 10.06.2011 | Winter-Spring-Summer |
