# Supplementary material for: Detection of QTL for traits related to adaptation to sub-optimal climatic conditions in chickens
Source: Genet Sel Evol. 2017 Apr 20;49:39. doi: 10.1186/s12711-017-0314-5 (PMC5399330; doi:10.1186/s12711-017-0314-5)
Supplement: Supplementary file 3 — Additional file 3: Figure S2. Variation in ambient temperature in the surrounding area of the experimental farm at the time of the experiment. [file 12711_2017_314_MOESM3_ESM.docx]

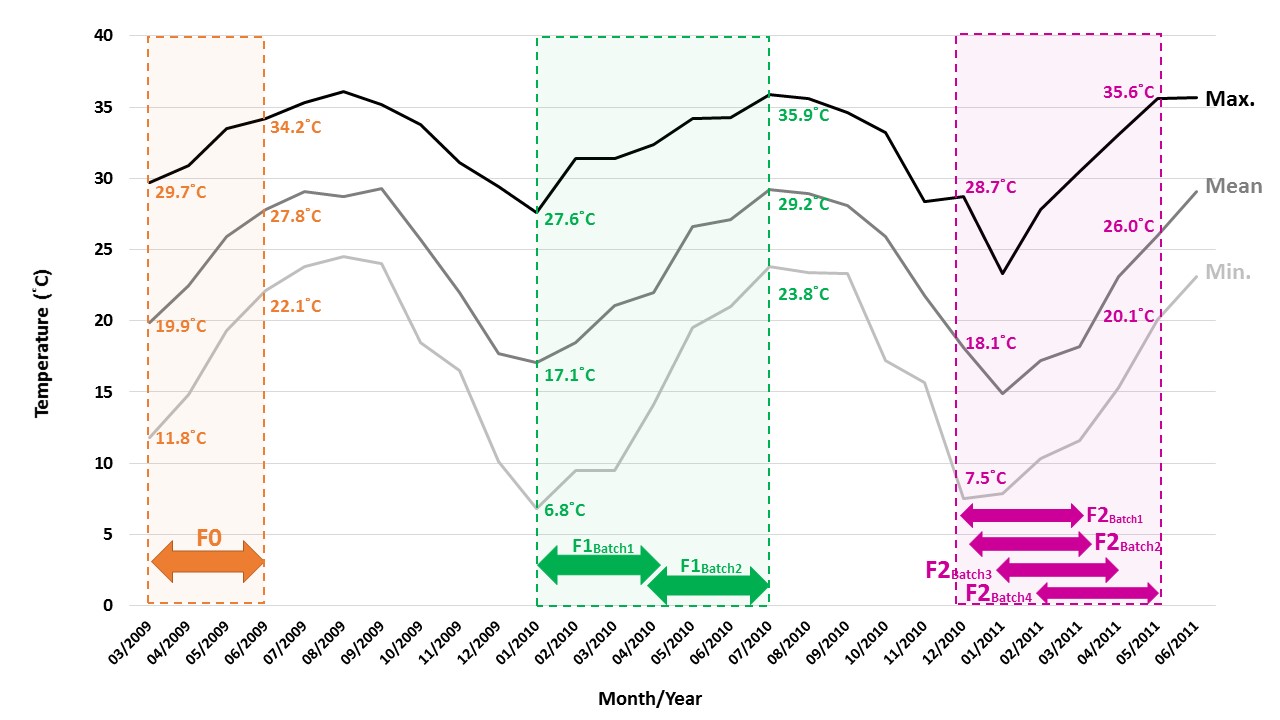


Fig. S2 The variation in ambient temperature in the surrounding area of the experimental farm at the time of the experiment
