# Supplementary material for: Detection of QTL for traits related to adaptation to sub-optimal climatic conditions in chickens
Source: Genet Sel Evol. 2017 Apr 20;49:39. doi: 10.1186/s12711-017-0314-5 (PMC5399330; doi:10.1186/s12711-017-0314-5)
Supplement: Supplementary file 7 — Additional file 7: Table S2. Results of the GWAS: list of SNPs showing 5% Bonferroni genome-wide significance for the recorded traits. [file 12711_2017_314_MOESM7_ESM.docx]

| Table S2 Results of the GWAS: list of SNPs showing 5 % Bonferroni genome-wide significance for the recorded traits | | | | | |
| --- | --- | --- | --- | --- | --- |
| **Trait** | **GGA** | **SNP** | **Pos(bp)** | **Nearest Gene** | **P-value** |
| BW04 | 24 | Gga_rs16197449 | 4145811 | LOC100857875 | 6.62E-07 |
| BW04 | 24 | GGaluGA192749 | 4201006 | - | 3.50E-06 |
| BW04 | 24 | GGaluGA192782 | 4247674 | - | 2.21E-06 |
| BW04 | 24 | GGaluGA192799 | 4286369 | - | 5.05E-07 |
| BW04 | 24 | Gga_rs15223001 | 4469987 | USP28 | 4.31E-06 |
| BW04 | 24 | Gga_rs14296323 | 4527463 | ZBTB16 | 2.43E-06 |
| BW04 | 24 | Gga_rs14297847 | 4934794 | - | 1.25E-06 |
| BW04 | 24 | GGaluGA193182 | 5030631 | - | 1.36E-06 |
| BW04 | 24 | Gga_rs16198299 | 5045556 | - | 1.05E-06 |
| BW04 | 24 | Gga_rs15224353 | 5255843 | - | 2.56E-06 |
| BW04 | 24 | Gga_rs14296647 | 5260488 | - | 1.09E-06 |
| BW04 | 24 | Gga_rs15224527 | 5316707 | - | 4.79E-07 |
| BW04 | 24 | Gga_rs14296677 | 5321244 | - | 1.34E-06 |
| BW04 | 39 | Gga_rs13817660 | 11113817 | GDNF | 2.20E-06 |
| BW04 | 39 | Gga_rs13800656 | 11327074 | EGFLAM | 1.36E-14 |
| BW04 | 39 | GGaluGA347965 | 11370132 | EGFLAM | 1.36E-14 |
| BW04 | 39 | Gga_rs14786514 | 11472405 | - | 2.59E-08 |
| BW04 | 39 | Gga_rs14786617 | 11562673 | OSMR | 7.08E-09 |
| BW04 | 39 | Gga_rs16781411 | 11673306 | FYB | 7.08E-09 |
| BW04 | 39 | Gga_rs14786725 | 11693740 | FYB | 7.08E-09 |
| BW04 | 39 | Gga_rs14786812 | 11788319 | - | 7.08E-09 |
| BW04 | 39 | Gga_rs14755855 | 12043692 | - | 7.08E-09 |
| BW04 | 39 | Gga_rs14755900 | 12085507 | - | 2.59E-15 |
| BW04 | 39 | Gga_rs14755968 | 12151053 | - | 2.59E-15 |
| BW04 | 39 | Gga_rs14756044 | 12262828 | PTGER4 | 2.59E-15 |
| BW04 | 39 | Gga_rs14756128 | 12347485 | - | 2.59E-15 |
| BW04 | 39 | Gga_rs14756165 | 12368483 | C7 | 2.59E-15 |
| BW04 | 39 | Gga_rs14756180 | 12389747 | C6 | 7.08E-09 |
| BW04 | 39 | Gga_rs14756234 | 12462388 | LOC100857889 | 2.59E-15 |
| BW04 | 39 | Gga_rs14756256 | 12479404 | LOC100857889 | 2.59E-15 |
| BW04 | 39 | Gga_rs14756284 | 12507150 | LOC100857889 | 2.59E-15 |
| BW04 | 39 | Gga_rs16102951 | 12906327 | - | 1.44E-06 |
| BW04 | 39 | Gga_rs14756703 | 12984581 | - | 1.44E-06 |
| BW04 | 39 | Gga_rs16763012 | 13179849 | NNT | 4.29E-06 |
| BW04 | 39 | Gga_rs14756923 | 13202902 | NNT | 4.29E-06 |
| BW04 | 39 | Gga_rs14757000 | 13288165 | - | 4.29E-06 |
| BW04 | 39 | Gga_rs16763062 | 13447769 | FGF10 | 4.29E-06 |
| BW04 | 39 | Gga_rs16103343 | 13591394 | - | 4.29E-06 |
| BW04 | 39 | GGaluGA348247 | 13611640 | - | 4.29E-06 |
| BW04 | 39 | Gga_rs13728807 | 13707137 | - | 1.27E-07 |
| BW04 | 39 | Gga_rs14757246 | 13714920 | - | 1.27E-07 |
| BW04 | 39 | Gga_rs14757289 | 13794465 | - | 1.27E-07 |
| BW04 | 39 | Gga_rs14757292 | 13812082 | - | 3.32E-06 |
| BW04 | 39 | Gga_rs16103436 | 13835031 | HCN1 | 1.27E-07 |
| BW04 | 39 | Gga_rs14757339 | 13977936 | HCN1 | 3.43E-08 |
| BW04 | 39 | GGaluGA348291 | 14030756 | HCN1 | 3.43E-08 |
| BW04 | 39 | Gga_rs13728832 | 14084602 | - | 3.43E-08 |
| BW04 | 39 | Gga_rs16103602 | 14189346 | LOC101748367 | 2.34E-12 |
| BW04 | 39 | Gga_rs16763234 | 14205194 | - | 8.26E-08 |
| BW04 | 39 | Gga_rs13728859 | 14260857 | PARP8 | 1.80E-07 |
| BW04 | 39 | GGaluGA348328 | 14328915 | - | 3.61E-07 |
| BW04 | 39 | Gga_rs16103781 | 14444488 | - | 3.61E-07 |
| BW04 | 39 | Gga_rs16763572 | 15196080 | ITGA2 | 2.94E-11 |
| BW04 | 39 | Gga_rs16104128 | 15674108 | ARL15 | 7.42E-07 |
| BW04 | 39 | Gga_rs14752031 | 16190785 | PPAP2A | 7.42E-07 |
| BW04 | 39 | Gga_rs13676590 | 16867867 | - | 1.71E-07 |
| BW04 | 39 | Gga_rs14752442 | 16892229 | GPBP1 | 4.09E-06 |
| BW04 | 39 | Gga_rs14752792 | 17445314 | RAB3C | 8.78E-07 |
| BW04 | 39 | Gga_rs14752843 | 17500236 | RAB3C | 4.09E-06 |
| BW04 | 39 | GGaluGA348716 | 17529244 | RAB3C | 4.09E-06 |
| BW04 | 39 | Gga_rs14752927 | 17612828 | PDE4D | 8.78E-07 |
| BW04 | 39 | GGaluGA348733 | 17687145 | PDE4D | 8.78E-07 |
| BW04 | 39 | Gga_rs14752983 | 17739555 | PDE4D | 8.33E-07 |
| BW08 | 24 | GGaluGA192799 | 4286369 | - | 3.03E-06 |
| BW08 | 24 | Gga_rs15223216 | 4535908 | ZBTB16 | 3.39E-06 |
| BW08 | 24 | Gga_rs15224353 | 5255843 | - | 2.18E-06 |
| BW08 | 24 | Gga_rs13724873 | 5302160 | LOC101750751 | 2.22E-06 |
| BW08 | 24 | Gga_rs15225342 | 5666441 | LOC101748511 | 4.23E-06 |
| BW08 | 24 | Gga_rs15225465 | 5687251 | LOC419782 | 3.04E-06 |
| BW08 | 24 | Gga_rs15226513 | 6019223 | - | 3.03E-06 |
| BW08 | 39 | Gga_rs14785130 | 9581584 | ADAMTS12 | 1.75E-06 |
| BW08 | 39 | Gga_rs14785179 | 9659034 | ADAMTS12 | 2.37E-06 |
| BW08 | 39 | Gga_rs14785203 | 9700700 | - | 2.37E-06 |
| BW08 | 39 | Gga_rs14785235 | 9744903 | - | 2.01E-06 |
| BW08 | 39 | Gga_rs14708266 | 9808980 | - | 6.62E-07 |
| BW08 | 39 | Gga_rs16130332 | 9833681 | RAI14 | 1.80E-06 |
| BW08 | 39 | Gga_rs16780726 | 9871880 | RAI14 | 1.80E-06 |
| BW08 | 39 | Gga_rs14785443 | 9975736 | - | 4.24E-06 |
| BW08 | 39 | GGaluGA347787 | 9995191 | - | 4.24E-06 |
| BW08 | 39 | Gga_rs16130424 | 10049151 | - | 4.24E-06 |
| BW08 | 39 | Gga_rs14731045 | 10187477 | SPEF2 | 2.51E-06 |
| BW08 | 39 | Gga_rs14731057 | 10212209 | - | 2.51E-06 |
| BW08 | 39 | Gga_rs16067654 | 10234193 | IL7R | 2.51E-06 |
| BW08 | 39 | GGaluGA347845 | 10361382 | NADKD1 | 4.21E-06 |
| BW08 | 39 | Gga_rs13817561 | 10463085 | - | 5.01E-07 |
| BW08 | 39 | Gga_rs14785793 | 10575355 | SLC1A3 | 5.01E-07 |
| BW08 | 39 | GGaluGA347920 | 10983124 | WDR70 | 1.21E-08 |
| BW08 | 39 | Gga_rs13817660 | 11113817 | GDNF | 4.22E-14 |
| BW08 | 39 | Gga_rs14786270 | 11214452 | - | 3.21E-06 |
| BW08 | 39 | Gga_rs13800656 | 11327074 | EGFLAM | 3.15E-42 |
| BW08 | 39 | GGaluGA347965 | 11370132 | EGFLAM | 3.15E-42 |
| BW08 | 39 | Gga_rs14786514 | 11472405 | - | 1.36E-20 |
| BW08 | 39 | GGaluGA347983 | 11500972 | - | 1.69E-11 |
| BW08 | 39 | GGaluGA347986 | 11508012 | LOC101751301 | 1.69E-11 |
| BW08 | 39 | Gga_rs14786617 | 11562673 | OSMR | 3.61E-22 |
| BW08 | 39 | GGaluGA348000 | 11576834 | RICTOR | 6.67E-10 |
| BW08 | 39 | Gga_rs14786654 | 11608187 | RICTOR | 6.67E-10 |
| BW08 | 39 | Gga_rs16781411 | 11673306 | FYB | 3.61E-22 |
| BW08 | 39 | Gga_rs14786725 | 11693740 | FYB | 3.61E-22 |
| BW08 | 39 | Gga_rs14786812 | 11788319 | - | 3.61E-22 |
| BW08 | 39 | Gga_rs14755855 | 12043692 | - | 3.61E-22 |
| BW08 | 39 | Gga_rs14755900 | 12085507 | - | 1.11E-44 |
| BW08 | 39 | Gga_rs16102386 | 12129543 | - | 1.98E-10 |
| BW08 | 39 | Gga_rs14755968 | 12151053 | - | 1.11E-44 |
| BW08 | 39 | GGaluGA348083 | 12205308 | - | 1.98E-10 |
| BW08 | 39 | Gga_rs13817336 | 12238730 | LOC101751864 | 1.98E-10 |
| BW08 | 39 | Gga_rs14756044 | 12262828 | PTGER4 | 1.11E-44 |
| BW08 | 39 | Gga_rs14756128 | 12347485 | - | 1.11E-44 |
| BW08 | 39 | Gga_rs14756165 | 12368483 | C7 | 1.11E-44 |
| BW08 | 39 | Gga_rs14756180 | 12389747 | C6 | 3.61E-22 |
| BW08 | 39 | Gga_rs14756234 | 12462388 | LOC100857889 | 1.11E-44 |
| BW08 | 39 | Gga_rs14756256 | 12479404 | LOC100857889 | 1.11E-44 |
| BW08 | 39 | Gga_rs14756284 | 12507150 | LOC100857889 | 1.11E-44 |
| BW08 | 39 | Gga_rs16102951 | 12906327 | - | 2.03E-13 |
| BW08 | 39 | Gga_rs14756703 | 12984581 | - | 2.03E-13 |
| BW08 | 39 | Gga_rs16763012 | 13179849 | NNT | 2.71E-09 |
| BW08 | 39 | Gga_rs14756923 | 13202902 | NNT | 2.71E-09 |
| BW08 | 39 | Gga_rs14756962 | 13221684 | - | 7.56E-12 |
| BW08 | 39 | Gga_rs14757000 | 13288165 | - | 2.71E-09 |
| BW08 | 39 | Gga_rs14757044 | 13376137 | - | 1.89E-10 |
| BW08 | 39 | Gga_rs16763062 | 13447769 | FGF10 | 2.71E-09 |
| BW08 | 39 | Gga_rs16103343 | 13591394 | - | 2.71E-09 |
| BW08 | 39 | GGaluGA348247 | 13611640 | - | 2.71E-09 |
| BW08 | 39 | Gga_rs13728807 | 13707137 | - | 2.29E-13 |
| BW08 | 39 | Gga_rs14757246 | 13714920 | - | 2.29E-13 |
| BW08 | 39 | Gga_rs14757289 | 13794465 | - | 2.29E-13 |
| BW08 | 39 | Gga_rs14757292 | 13812082 | - | 1.91E-10 |
| BW08 | 39 | Gga_rs16103436 | 13835031 | HCN1 | 2.29E-13 |
| BW08 | 39 | Gga_rs14757339 | 13977936 | HCN1 | 1.23E-18 |
| BW08 | 39 | Gga_rs14757367 | 14020461 | HCN1 | 2.70E-11 |
| BW08 | 39 | GGaluGA348291 | 14030756 | HCN1 | 1.23E-18 |
| BW08 | 39 | Gga_rs13728832 | 14084602 | - | 1.23E-18 |
| BW08 | 39 | Gga_rs16103602 | 14189346 | LOC101748367 | 2.65E-35 |
| BW08 | 39 | Gga_rs16763234 | 14205194 | - | 9.69E-18 |
| BW08 | 39 | Gga_rs13728859 | 14260857 | PARP8 | 1.27E-16 |
| BW08 | 39 | GGaluGA348328 | 14328915 | - | 1.17E-16 |
| BW08 | 39 | Gga_rs16103781 | 14444488 | - | 1.17E-16 |
| BW08 | 39 | Gga_rs14757660 | 14468384 | - | 1.40E-09 |
| BW08 | 39 | Gga_rs16103818 | 14584514 | - | 9.96E-09 |
| BW08 | 39 | Gga_rs16763394 | 14658324 | - | 2.35E-06 |
| BW08 | 39 | Gga_rs14757750 | 14738731 | - | 9.96E-09 |
| BW08 | 39 | Gga_rs14757759 | 14765320 | - | 4.67E-07 |
| BW08 | 39 | Gga_rs16103911 | 14851712 | - | 9.96E-09 |
| BW08 | 39 | Gga_rs14757836 | 14876950 | - | 9.96E-09 |
| BW08 | 39 | Gga_rs14757875 | 15142140 | ITGA1 | 4.67E-07 |
| BW08 | 39 | Gga_rs16763572 | 15196080 | ITGA2 | 3.57E-33 |
| BW08 | 39 | Gga_rs16763589 | 15222404 | ITGA2 | 2.35E-06 |
| BW08 | 39 | Gga_rs16103980 | 15244465 | ITGA2 | 9.96E-09 |
| BW08 | 39 | Gga_rs14757928 | 15257934 | MOCS2 | 9.96E-09 |
| BW08 | 39 | Gga_rs16104128 | 15674108 | ARL15 | 1.51E-11 |
| BW08 | 39 | Gga_rs14758352 | 15898298 | LOC425372 | 4.01E-08 |
| BW08 | 39 | Gga_rs14740460 | 15956247 | - | 4.01E-08 |
| BW08 | 39 | Gga_rs14752031 | 16190785 | PPAP2A | 1.51E-11 |
| BW08 | 39 | GGaluGA348537 | 16520460 | - | 3.06E-07 |
| BW08 | 39 | Gga_rs14752383 | 16798487 | MAP3K1 | 5.59E-07 |
| BW08 | 39 | Gga_rs13676590 | 16867867 | - | 1.04E-25 |
| BW08 | 39 | Gga_rs14752442 | 16892229 | GPBP1 | 2.76E-10 |
| BW08 | 39 | Gga_rs16760371 | 16912728 | GPBP1 | 1.76E-10 |
| BW08 | 39 | Gga_rs13676607 | 16958770 | - | 1.51E-06 |
| BW08 | 39 | Gga_rs14752792 | 17445314 | RAB3C | 5.41E-23 |
| BW08 | 39 | Gga_rs14752843 | 17500236 | RAB3C | 2.76E-10 |
| BW08 | 39 | GGaluGA348716 | 17529244 | RAB3C | 2.76E-10 |
| BW08 | 39 | Gga_rs14752927 | 17612828 | PDE4D | 5.41E-23 |
| BW08 | 39 | GGaluGA348733 | 17687145 | PDE4D | 5.41E-23 |
| BW08 | 39 | Gga_rs14752983 | 17739555 | PDE4D | 8.18E-23 |
| BW08 | 39 | GGaluGA348796 | 18278538 | - | 2.77E-10 |
| BW08 | 39 | GGaluGA348802 | 18308486 | ERCC8 | 2.77E-10 |
| BW08 | 39 | Gga_rs14753237 | 18354247 | NDUFAF2 | 2.77E-10 |
| BW08 | 39 | GGaluGA348847 | 18577655 | ZSWIM6 | 3.38E-20 |
| BW08 | 39 | GGaluGA348898 | 18816948 | KIF2A | 2.72E-19 |
| BW08 | 39 | Gga_rs16100247 | 19021102 | - | 9.62E-10 |
| BW08 | 39 | GGaluGA348948 | 19186417 | - | 2.72E-19 |
| BW08 | 39 | GGaluGA348980 | 19286706 | - | 2.72E-19 |
| BW08 | 39 | GGaluGA348985 | 19313055 | - | 9.62E-10 |
| BW08 | 39 | Gga_rs16100722 | 19834059 | ADAMTS6 | 1.16E-09 |
| BW08 | 39 | GGaluGA349087 | 19992764 | PPWD1 | 8.20E-19 |
| BW08 | 39 | Gga_rs14754344 | 20015776 | TRIM23 | 8.20E-19 |
| BW08 | 39 | Gga_rs13676932 | 20151804 | ERBB2IP | 1.16E-09 |
| BW08 | 39 | Gga_rs14754805 | 20487433 | MAST4 | 1.23E-09 |
| BW08 | 39 | Gga_rs16101219 | 20553419 | MAST4 | 1.44E-18 |
| BW08 | 39 | GGaluGA349195 | 20702054 | MAST4 | 1.15E-09 |
| BW08 | 39 | Gga_rs13677070 | 21139710 | - | 3.55E-15 |
| BW08 | 39 | GGaluGA349294 | 21146543 | - | 3.55E-15 |
| BW08 | 39 | Gga_rs14755437 | 21359710 | CDK7 | 1.63E-08 |
| BW08 | 39 | GGaluGA349348 | 21363270 | CDK7 | 1.63E-08 |
| BW08 | 39 | GGaluGA349353 | 21409504 | SERINC5 | 1.63E-08 |
| BW08 | 39 | GGaluGA349472 | 21899444 | DMGDH | 3.20E-07 |
| BW08 | 39 | GGaluGA349476 | 21909031 | - | 7.37E-08 |
| BW08 | 39 | GGaluGA349488 | 21954151 | ARSB | 2.29E-07 |
| BW0804 | 39 | Gga_rs16129626 | 9088090 | MTMR12 | 2.84E-06 |
| BW0804 | 39 | GGaluGA347637 | 9095810 | - | 2.42E-06 |
| BW0804 | 39 | Gga_rs14784876 | 9131966 | ZFR | 2.42E-06 |
| BW0804 | 39 | GGaluGA347642 | 9146754 | - | 2.42E-06 |
| BW0804 | 39 | Gga_rs14784984 | 9328599 | - | 1.13E-06 |
| BW0804 | 39 | Gga_rs14785019 | 9365275 | - | 6.33E-07 |
| BW0804 | 39 | Gga_rs16129856 | 9391651 | - | 6.33E-07 |
| BW0804 | 39 | Gga_rs13769832 | 9472650 | TARS | 6.33E-07 |
| BW0804 | 39 | Gga_rs14785130 | 9581584 | ADAMTS12 | 3.04E-07 |
| BW0804 | 39 | Gga_rs14785179 | 9659034 | ADAMTS12 | 5.84E-07 |
| BW0804 | 39 | Gga_rs14785203 | 9700700 | - | 5.84E-07 |
| BW0804 | 39 | Gga_rs14785235 | 9744903 | - | 3.52E-07 |
| BW0804 | 39 | Gga_rs14708266 | 9808980 | - | 1.29E-07 |
| BW0804 | 39 | Gga_rs16130332 | 9833681 | RAI14 | 2.60E-07 |
| BW0804 | 39 | Gga_rs16780726 | 9871880 | RAI14 | 2.60E-07 |
| BW0804 | 39 | Gga_rs14785443 | 9975736 | - | 4.71E-07 |
| BW0804 | 39 | GGaluGA347787 | 9995191 | - | 4.71E-07 |
| BW0804 | 39 | GGaluGA347788 | 10005474 | - | 3.83E-06 |
| BW0804 | 39 | Gga_rs16130424 | 10049151 | - | 4.71E-07 |
| BW0804 | 39 | Gga_rs16780806 | 10065018 | - | 5.33E-07 |
| BW0804 | 39 | Gga_rs14731045 | 10187477 | SPEF2 | 3.14E-07 |
| BW0804 | 39 | Gga_rs14731057 | 10212209 | - | 3.14E-07 |
| BW0804 | 39 | Gga_rs16067654 | 10234193 | IL7R | 3.14E-07 |
| BW0804 | 39 | GGaluGA347845 | 10361382 | NADKD1 | 8.97E-07 |
| BW0804 | 39 | Gga_rs13817561 | 10463085 | - | 8.33E-08 |
| BW0804 | 39 | Gga_rs14785793 | 10575355 | SLC1A3 | 8.33E-08 |
| BW0804 | 39 | GGaluGA347920 | 10983124 | WDR70 | 1.95E-09 |
| BW0804 | 39 | Gga_rs13817660 | 11113817 | GDNF | 6.50E-15 |
| BW0804 | 39 | Gga_rs14786270 | 11214452 | - | 4.06E-06 |
| BW0804 | 39 | Gga_rs13800656 | 11327074 | EGFLAM | 8.54E-45 |
| BW0804 | 39 | GGaluGA347965 | 11370132 | EGFLAM | 8.54E-45 |
| BW0804 | 39 | Gga_rs14786514 | 11472405 | - | 1.11E-20 |
| BW0804 | 39 | GGaluGA347983 | 11500972 | - | 2.81E-12 |
| BW0804 | 39 | GGaluGA347986 | 11508012 | LOC101751301 | 2.81E-12 |
| BW0804 | 39 | Gga_rs14786617 | 11562673 | OSMR | 3.33E-22 |
| BW0804 | 39 | GGaluGA348000 | 11576834 | RICTOR | 1.25E-10 |
| BW0804 | 39 | Gga_rs14786654 | 11608187 | RICTOR | 1.25E-10 |
| BW0804 | 39 | Gga_rs16781411 | 11673306 | FYB | 3.33E-22 |
| BW0804 | 39 | Gga_rs14786725 | 11693740 | FYB | 3.33E-22 |
| BW0804 | 39 | Gga_rs14786812 | 11788319 | - | 3.33E-22 |
| BW0804 | 39 | Gga_rs14755855 | 12043692 | - | 3.33E-22 |
| BW0804 | 39 | Gga_rs14755900 | 12085507 | - | 1.74E-47 |
| BW0804 | 39 | Gga_rs16102386 | 12129543 | - | 5.25E-11 |
| BW0804 | 39 | Gga_rs14755968 | 12151053 | - | 1.74E-47 |
| BW0804 | 39 | GGaluGA348083 | 12205308 | - | 5.25E-11 |
| BW0804 | 39 | Gga_rs13817336 | 12238730 | LOC101751864 | 5.25E-11 |
| BW0804 | 39 | Gga_rs14756044 | 12262828 | PTGER4 | 1.74E-47 |
| BW0804 | 39 | Gga_rs14756128 | 12347485 | - | 1.74E-47 |
| BW0804 | 39 | Gga_rs14756165 | 12368483 | C7 | 1.74E-47 |
| BW0804 | 39 | Gga_rs14756180 | 12389747 | C6 | 3.33E-22 |
| BW0804 | 39 | Gga_rs14756234 | 12462388 | LOC100857889 | 1.74E-47 |
| BW0804 | 39 | Gga_rs14756256 | 12479404 | LOC100857889 | 1.74E-47 |
| BW0804 | 39 | Gga_rs14756284 | 12507150 | LOC100857889 | 1.74E-47 |
| BW0804 | 39 | Gga_rs16102951 | 12906327 | - | 6.55E-14 |
| BW0804 | 39 | Gga_rs14756703 | 12984581 | - | 6.55E-14 |
| BW0804 | 39 | Gga_rs16763012 | 13179849 | NNT | 3.82E-08 |
| BW0804 | 39 | Gga_rs14756923 | 13202902 | NNT | 3.82E-08 |
| BW0804 | 39 | Gga_rs14756962 | 13221684 | - | 3.49E-13 |
| BW0804 | 39 | Gga_rs14757000 | 13288165 | - | 3.82E-08 |
| BW0804 | 39 | Gga_rs14757044 | 13376137 | - | 4.48E-11 |
| BW0804 | 39 | Gga_rs16763062 | 13447769 | FGF10 | 3.82E-08 |
| BW0804 | 39 | Gga_rs16103343 | 13591394 | - | 3.82E-08 |
| BW0804 | 39 | GGaluGA348247 | 13611640 | - | 3.82E-08 |
| BW0804 | 39 | Gga_rs13728807 | 13707137 | - | 2.83E-13 |
| BW0804 | 39 | Gga_rs14757246 | 13714920 | - | 2.83E-13 |
| BW0804 | 39 | Gga_rs14757289 | 13794465 | - | 2.83E-13 |
| BW0804 | 39 | Gga_rs14757292 | 13812082 | - | 1.13E-10 |
| BW0804 | 39 | Gga_rs16103436 | 13835031 | HCN1 | 2.83E-13 |
| BW0804 | 39 | Gga_rs14757339 | 13977936 | HCN1 | 2.37E-18 |
| BW0804 | 39 | Gga_rs14757367 | 14020461 | HCN1 | 8.72E-12 |
| BW0804 | 39 | GGaluGA348291 | 14030756 | HCN1 | 2.37E-18 |
| BW0804 | 39 | Gga_rs13728832 | 14084602 | - | 2.37E-18 |
| BW0804 | 39 | Gga_rs16103602 | 14189346 | LOC101748367 | 1.18E-38 |
| BW0804 | 39 | Gga_rs16763234 | 14205194 | - | 1.64E-17 |
| BW0804 | 39 | Gga_rs13728859 | 14260857 | PARP8 | 1.96E-16 |
| BW0804 | 39 | GGaluGA348328 | 14328915 | - | 1.20E-16 |
| BW0804 | 39 | Gga_rs16103781 | 14444488 | - | 1.20E-16 |
| BW0804 | 39 | Gga_rs14757660 | 14468384 | - | 4.09E-10 |
| BW0804 | 39 | Gga_rs16103818 | 14584514 | - | 2.75E-09 |
| BW0804 | 39 | Gga_rs16763394 | 14658324 | - | 9.99E-07 |
| BW0804 | 39 | Gga_rs14757750 | 14738731 | - | 2.75E-09 |
| BW0804 | 39 | Gga_rs14757759 | 14765320 | - | 8.38E-08 |
| BW0804 | 39 | Gga_rs16103911 | 14851712 | - | 2.75E-09 |
| BW0804 | 39 | Gga_rs14757836 | 14876950 | - | 2.75E-09 |
| BW0804 | 39 | Gga_rs14757875 | 15142140 | ITGA1 | 8.38E-08 |
| BW0804 | 39 | Gga_rs16763572 | 15196080 | ITGA2 | 9.51E-37 |
| BW0804 | 39 | Gga_rs16763589 | 15222404 | ITGA2 | 9.99E-07 |
| BW0804 | 39 | Gga_rs16103980 | 15244465 | ITGA2 | 2.75E-09 |
| BW0804 | 39 | Gga_rs14757928 | 15257934 | MOCS2 | 2.75E-09 |
| BW0804 | 39 | Gga_rs16104128 | 15674108 | ARL15 | 7.26E-11 |
| BW0804 | 39 | Gga_rs14758352 | 15898298 | LOC425372 | 5.20E-07 |
| BW0804 | 39 | Gga_rs14740460 | 15956247 | - | 5.20E-07 |
| BW0804 | 39 | Gga_rs14752031 | 16190785 | PPAP2A | 7.26E-11 |
| BW0804 | 39 | GGaluGA348537 | 16520460 | - | 2.82E-06 |
| BW0804 | 39 | Gga_rs14752383 | 16798487 | MAP3K1 | 8.13E-09 |
| BW0804 | 39 | Gga_rs13676590 | 16867867 | - | 2.50E-30 |
| BW0804 | 39 | Gga_rs14752442 | 16892229 | GPBP1 | 9.08E-10 |
| BW0804 | 39 | Gga_rs16760371 | 16912728 | GPBP1 | 4.75E-10 |
| BW0804 | 39 | Gga_rs13676607 | 16958770 | - | 2.26E-08 |
| BW0804 | 39 | Gga_rs14752792 | 17445314 | RAB3C | 8.12E-27 |
| BW0804 | 39 | Gga_rs14752843 | 17500236 | RAB3C | 9.08E-10 |
| BW0804 | 39 | GGaluGA348716 | 17529244 | RAB3C | 9.08E-10 |
| BW0804 | 39 | Gga_rs14752927 | 17612828 | PDE4D | 8.12E-27 |
| BW0804 | 39 | GGaluGA348730 | 17651744 | PDE4D | 1.75E-06 |
| BW0804 | 39 | GGaluGA348733 | 17687145 | PDE4D | 8.12E-27 |
| BW0804 | 39 | Gga_rs14752983 | 17739555 | PDE4D | 1.71E-26 |
| BW0804 | 39 | Gga_rs16099588 | 18161143 | - | 1.75E-06 |
| BW0804 | 39 | GGaluGA348796 | 18278538 | - | 2.35E-11 |
| BW0804 | 39 | GGaluGA348802 | 18308486 | ERCC8 | 2.35E-11 |
| BW0804 | 39 | Gga_rs14753237 | 18354247 | NDUFAF2 | 2.35E-11 |
| BW0804 | 39 | GGaluGA348847 | 18577655 | ZSWIM6 | 3.37E-24 |
| BW0804 | 39 | GGaluGA348898 | 18816948 | KIF2A | 3.39E-23 |
| BW0804 | 39 | Gga_rs16100247 | 19021102 | - | 1.01E-10 |
| BW0804 | 39 | GGaluGA348948 | 19186417 | - | 3.39E-23 |
| BW0804 | 39 | Gga_rs16100380 | 19270618 | - | 2.75E-07 |
| BW0804 | 39 | GGaluGA348980 | 19286706 | - | 3.39E-23 |
| BW0804 | 39 | GGaluGA348985 | 19313055 | - | 1.01E-10 |
| BW0804 | 39 | Gga_rs16100540 | 19491611 | RNF180 | 2.75E-07 |
| BW0804 | 39 | Gga_rs16100722 | 19834059 | ADAMTS6 | 1.20E-10 |
| BW0804 | 39 | GGaluGA349087 | 19992764 | PPWD1 | 1.47E-22 |
| BW0804 | 39 | Gga_rs14754344 | 20015776 | TRIM23 | 1.47E-22 |
| BW0804 | 39 | Gga_rs13676932 | 20151804 | ERBB2IP | 1.20E-10 |
| BW0804 | 39 | Gga_rs14754805 | 20487433 | MAST4 | 9.52E-11 |
| BW0804 | 39 | Gga_rs16101219 | 20553419 | MAST4 | 2.18E-22 |
| BW0804 | 39 | GGaluGA349195 | 20702054 | MAST4 | 1.85E-10 |
| BW0804 | 39 | Gga_rs14755141 | 20899496 | - | 5.72E-08 |
| BW0804 | 39 | Gga_rs14755151 | 20917367 | - | 5.72E-08 |
| BW0804 | 39 | Gga_rs13677070 | 21139710 | - | 9.00E-18 |
| BW0804 | 39 | GGaluGA349294 | 21146543 | - | 9.00E-18 |
| BW0804 | 39 | Gga_rs16101716 | 21183007 | - | 1.23E-07 |
| BW0804 | 39 | Gga_rs14755437 | 21359710 | CDK7 | 6.58E-08 |
| BW0804 | 39 | GGaluGA349348 | 21363270 | CDK7 | 6.58E-08 |
| BW0804 | 39 | GGaluGA349353 | 21409504 | SERINC5 | 6.58E-08 |
| BW0804 | 39 | GGaluGA349472 | 21899444 | DMGDH | 1.02E-06 |
| BW0804 | 39 | GGaluGA349476 | 21909031 | - | 1.75E-09 |
| BW0804 | 39 | GGaluGA349488 | 21954151 | ARSB | 4.39E-07 |
| BW12 | 39 | GGaluGA347920 | 10983124 | WDR70 | 1.01E-06 |
| BW12 | 39 | Gga_rs13817660 | 11113817 | GDNF | 1.33E-09 |
| BW12 | 39 | Gga_rs13800656 | 11327074 | EGFLAM | 1.76E-30 |
| BW12 | 39 | GGaluGA347965 | 11370132 | EGFLAM | 1.76E-30 |
| BW12 | 39 | Gga_rs14786514 | 11472405 | - | 9.39E-16 |
| BW12 | 39 | GGaluGA347983 | 11500972 | - | 1.78E-08 |
| BW12 | 39 | GGaluGA347986 | 11508012 | LOC101751301 | 1.78E-08 |
| BW12 | 39 | Gga_rs14786617 | 11562673 | OSMR | 1.67E-17 |
| BW12 | 39 | GGaluGA348000 | 11576834 | RICTOR | 3.62E-07 |
| BW12 | 39 | Gga_rs14786654 | 11608187 | RICTOR | 3.62E-07 |
| BW12 | 39 | Gga_rs16781411 | 11673306 | FYB | 1.67E-17 |
| BW12 | 39 | Gga_rs14786725 | 11693740 | FYB | 1.67E-17 |
| BW12 | 39 | Gga_rs14786812 | 11788319 | - | 1.67E-17 |
| BW12 | 39 | Gga_rs14755855 | 12043692 | - | 1.67E-17 |
| BW12 | 39 | Gga_rs14755900 | 12085507 | - | 6.56E-33 |
| BW12 | 39 | Gga_rs16102386 | 12129543 | - | 1.19E-08 |
| BW12 | 39 | Gga_rs14755968 | 12151053 | - | 6.56E-33 |
| BW12 | 39 | GGaluGA348083 | 12205308 | - | 1.19E-08 |
| BW12 | 39 | Gga_rs13817336 | 12238730 | LOC101751864 | 1.19E-08 |
| BW12 | 39 | Gga_rs14756044 | 12262828 | PTGER4 | 6.56E-33 |
| BW12 | 39 | Gga_rs14756128 | 12347485 | - | 6.56E-33 |
| BW12 | 39 | Gga_rs14756165 | 12368483 | C7 | 6.56E-33 |
| BW12 | 39 | Gga_rs14756180 | 12389747 | C6 | 1.67E-17 |
| BW12 | 39 | Gga_rs14756234 | 12462388 | LOC100857889 | 6.56E-33 |
| BW12 | 39 | Gga_rs14756256 | 12479404 | LOC100857889 | 6.56E-33 |
| BW12 | 39 | Gga_rs14756284 | 12507150 | LOC100857889 | 6.56E-33 |
| BW12 | 39 | Gga_rs16102951 | 12906327 | - | 1.90E-10 |
| BW12 | 39 | Gga_rs14756703 | 12984581 | - | 1.90E-10 |
| BW12 | 39 | Gga_rs16763012 | 13179849 | NNT | 1.29E-07 |
| BW12 | 39 | Gga_rs14756923 | 13202902 | NNT | 1.29E-07 |
| BW12 | 39 | Gga_rs14756962 | 13221684 | - | 7.33E-09 |
| BW12 | 39 | Gga_rs14757000 | 13288165 | - | 1.29E-07 |
| BW12 | 39 | Gga_rs14757044 | 13376137 | - | 8.43E-09 |
| BW12 | 39 | Gga_rs16763062 | 13447769 | FGF10 | 1.29E-07 |
| BW12 | 39 | Gga_rs16103343 | 13591394 | - | 1.29E-07 |
| BW12 | 39 | GGaluGA348247 | 13611640 | - | 1.29E-07 |
| BW12 | 39 | Gga_rs13728807 | 13707137 | - | 1.72E-10 |
| BW12 | 39 | Gga_rs14757246 | 13714920 | - | 1.72E-10 |
| BW12 | 39 | Gga_rs14757289 | 13794465 | - | 1.72E-10 |
| BW12 | 39 | Gga_rs14757292 | 13812082 | - | 9.57E-09 |
| BW12 | 39 | Gga_rs16103436 | 13835031 | HCN1 | 1.72E-10 |
| BW12 | 39 | Gga_rs14757339 | 13977936 | HCN1 | 1.78E-14 |
| BW12 | 39 | Gga_rs14757367 | 14020461 | HCN1 | 5.93E-09 |
| BW12 | 39 | GGaluGA348291 | 14030756 | HCN1 | 1.78E-14 |
| BW12 | 39 | Gga_rs13728832 | 14084602 | - | 1.78E-14 |
| BW12 | 39 | Gga_rs16103602 | 14189346 | LOC101748367 | 3.16E-27 |
| BW12 | 39 | Gga_rs16763234 | 14205194 | - | 1.87E-14 |
| BW12 | 39 | Gga_rs13728859 | 14260857 | PARP8 | 7.29E-14 |
| BW12 | 39 | GGaluGA348328 | 14328915 | - | 1.04E-13 |
| BW12 | 39 | Gga_rs16103781 | 14444488 | - | 1.04E-13 |
| BW12 | 39 | Gga_rs14757660 | 14468384 | - | 2.95E-08 |
| BW12 | 39 | Gga_rs16103818 | 14584514 | - | 9.86E-08 |
| BW12 | 39 | Gga_rs14757750 | 14738731 | - | 9.86E-08 |
| BW12 | 39 | Gga_rs14757759 | 14765320 | - | 9.21E-07 |
| BW12 | 39 | Gga_rs16103911 | 14851712 | - | 9.86E-08 |
| BW12 | 39 | Gga_rs14757836 | 14876950 | - | 9.86E-08 |
| BW12 | 39 | Gga_rs14757875 | 15142140 | ITGA1 | 9.21E-07 |
| BW12 | 39 | Gga_rs16763572 | 15196080 | ITGA2 | 8.64E-26 |
| BW12 | 39 | Gga_rs16103980 | 15244465 | ITGA2 | 9.86E-08 |
| BW12 | 39 | Gga_rs14757928 | 15257934 | MOCS2 | 9.86E-08 |
| BW12 | 39 | Gga_rs16104128 | 15674108 | ARL15 | 5.57E-10 |
| BW12 | 39 | Gga_rs14758352 | 15898298 | LOC425372 | 1.07E-06 |
| BW12 | 39 | Gga_rs14740460 | 15956247 | - | 1.07E-06 |
| BW12 | 39 | Gga_rs14752031 | 16190785 | PPAP2A | 5.57E-10 |
| BW12 | 39 | GGaluGA348537 | 16520460 | - | 4.57E-06 |
| BW12 | 39 | Gga_rs13676590 | 16867867 | - | 4.80E-20 |
| BW12 | 39 | Gga_rs14752442 | 16892229 | GPBP1 | 5.30E-09 |
| BW12 | 39 | Gga_rs16760371 | 16912728 | GPBP1 | 1.23E-08 |
| BW12 | 39 | Gga_rs14752792 | 17445314 | RAB3C | 1.44E-18 |
| BW12 | 39 | Gga_rs14752843 | 17500236 | RAB3C | 5.30E-09 |
| BW12 | 39 | GGaluGA348716 | 17529244 | RAB3C | 5.30E-09 |
| BW12 | 39 | Gga_rs14752927 | 17612828 | PDE4D | 1.44E-18 |
| BW12 | 39 | Gga_rs16099374 | 17675349 | PDE4D | 2.99E-06 |
| BW12 | 39 | GGaluGA348733 | 17687145 | PDE4D | 1.44E-18 |
| BW12 | 39 | Gga_rs16099440 | 17736692 | PDE4D | 2.99E-06 |
| BW12 | 39 | Gga_rs14752983 | 17739555 | PDE4D | 1.47E-18 |
| BW12 | 39 | Gga_rs16760787 | 17841753 | PDE4D | 2.99E-06 |
| BW12 | 39 | Gga_rs14753047 | 17879842 | PDE4D | 2.33E-06 |
| BW12 | 39 | Gga_rs16760857 | 18010099 | PDE4D | 2.99E-06 |
| BW12 | 39 | Gga_rs16099536 | 18091292 | - | 2.99E-06 |
| BW12 | 39 | Gga_rs16099639 | 18181771 | - | 2.99E-06 |
| BW12 | 39 | GGaluGA348796 | 18278538 | - | 9.58E-09 |
| BW12 | 39 | GGaluGA348802 | 18308486 | ERCC8 | 9.58E-09 |
| BW12 | 39 | Gga_rs14753237 | 18354247 | NDUFAF2 | 9.58E-09 |
| BW12 | 39 | GGaluGA348847 | 18577655 | ZSWIM6 | 2.24E-15 |
| BW12 | 39 | GGaluGA348898 | 18816948 | KIF2A | 9.55E-14 |
| BW12 | 39 | Gga_rs16100247 | 19021102 | - | 6.44E-09 |
| BW12 | 39 | GGaluGA348948 | 19186417 | - | 9.55E-14 |
| BW12 | 39 | GGaluGA348980 | 19286706 | - | 9.55E-14 |
| BW12 | 39 | GGaluGA348985 | 19313055 | - | 6.44E-09 |
| BW12 | 39 | Gga_rs16100722 | 19834059 | ADAMTS6 | 7.85E-09 |
| BW12 | 39 | GGaluGA349087 | 19992764 | PPWD1 | 2.25E-13 |
| BW12 | 39 | Gga_rs14754344 | 20015776 | TRIM23 | 2.25E-13 |
| BW12 | 39 | Gga_rs13676932 | 20151804 | ERBB2IP | 7.85E-09 |
| BW12 | 39 | Gga_rs14754805 | 20487433 | MAST4 | 7.76E-09 |
| BW12 | 39 | Gga_rs16101219 | 20553419 | MAST4 | 2.64E-13 |
| BW12 | 39 | GGaluGA349195 | 20702054 | MAST4 | 7.81E-09 |
| BW12 | 39 | Gga_rs13677070 | 21139710 | - | 2.90E-10 |
| BW12 | 39 | GGaluGA349294 | 21146543 | - | 2.90E-10 |
| BW1208 | 39 | Gga_rs13800656 | 11327074 | EGFLAM | 1.05E-06 |
| BW1208 | 39 | GGaluGA347965 | 11370132 | EGFLAM | 1.05E-06 |
| BW1208 | 39 | Gga_rs14755900 | 12085507 | - | 2.66E-07 |
| BW1208 | 39 | Gga_rs14755968 | 12151053 | - | 2.66E-07 |
| BW1208 | 39 | Gga_rs14756044 | 12262828 | PTGER4 | 2.66E-07 |
| BW1208 | 39 | Gga_rs14756128 | 12347485 | - | 2.66E-07 |
| BW1208 | 39 | Gga_rs14756165 | 12368483 | C7 | 2.66E-07 |
| BW1208 | 39 | Gga_rs14756234 | 12462388 | LOC100857889 | 2.66E-07 |
| BW1208 | 39 | Gga_rs14756256 | 12479404 | LOC100857889 | 2.66E-07 |
| BW1208 | 39 | Gga_rs14756284 | 12507150 | LOC100857889 | 2.66E-07 |
| BW1208 | 39 | Gga_rs16103602 | 14189346 | LOC101748367 | 6.75E-07 |
| BW1208 | 39 | Gga_rs16763572 | 15196080 | ITGA2 | 8.29E-07 |
| BW1208 | 39 | Gga_rs13676590 | 16867867 | - | 4.20E-06 |
| BW1208 | 39 | Gga_rs14752792 | 17445314 | RAB3C | 4.12E-06 |
| BW1208 | 39 | Gga_rs14752927 | 17612828 | PDE4D | 4.12E-06 |
| BW1208 | 39 | GGaluGA348733 | 17687145 | PDE4D | 4.12E-06 |
| BW1208 | 39 | Gga_rs14752983 | 17739555 | PDE4D | 3.71E-06 |
| BW16 | 39 | Gga_rs14695654 | 4646914 | - | 1.12E-06 |
| BW16 | 39 | Gga_rs13781756 | 5683707 | CELF4 | 1.36E-06 |
| BW16 | 39 | Gga_rs16687179 | 5909968 | CELF4 | 1.36E-06 |
| BW16 | 39 | Gga_rs14689250 | 5980966 | CELF4 | 1.36E-06 |
| BW16 | 39 | Gga_rs14689275 | 6023957 | CELF4 | 1.29E-06 |
| BW16 | 39 | Gga_rs16129626 | 9088090 | MTMR12 | 2.85E-06 |
| BW16 | 39 | Gga_rs14785130 | 9581584 | ADAMTS12 | 4.14E-06 |
| BW16 | 39 | Gga_rs14785179 | 9659034 | ADAMTS12 | 2.97E-06 |
| BW16 | 39 | Gga_rs14785203 | 9700700 | - | 2.97E-06 |
| BW16 | 39 | Gga_rs14785235 | 9744903 | - | 2.86E-06 |
| BW16 | 39 | Gga_rs14708266 | 9808980 | - | 9.02E-07 |
| BW16 | 39 | Gga_rs16130332 | 9833681 | RAI14 | 7.91E-07 |
| BW16 | 39 | Gga_rs16780726 | 9871880 | RAI14 | 7.91E-07 |
| BW16 | 39 | Gga_rs14785443 | 9975736 | - | 1.50E-06 |
| BW16 | 39 | GGaluGA347787 | 9995191 | - | 1.50E-06 |
| BW16 | 39 | Gga_rs16130424 | 10049151 | - | 1.50E-06 |
| BW16 | 39 | Gga_rs16780806 | 10065018 | - | 1.19E-06 |
| BW16 | 39 | Gga_rs14731045 | 10187477 | SPEF2 | 9.80E-07 |
| BW16 | 39 | Gga_rs14731057 | 10212209 | - | 9.80E-07 |
| BW16 | 39 | Gga_rs16067654 | 10234193 | IL7R | 9.80E-07 |
| BW16 | 39 | GGaluGA347845 | 10361382 | NADKD1 | 9.13E-07 |
| BW16 | 39 | Gga_rs13817561 | 10463085 | - | 9.15E-08 |
| BW16 | 39 | Gga_rs14785793 | 10575355 | SLC1A3 | 9.15E-08 |
| BW16 | 39 | GGaluGA347920 | 10983124 | WDR70 | 3.79E-09 |
| BW16 | 39 | Gga_rs13817660 | 11113817 | GDNF | 7.53E-15 |
| BW16 | 39 | Gga_rs13800656 | 11327074 | EGFLAM | 4.73E-37 |
| BW16 | 39 | GGaluGA347965 | 11370132 | EGFLAM | 4.73E-37 |
| BW16 | 39 | Gga_rs14786514 | 11472405 | - | 4.73E-18 |
| BW16 | 39 | GGaluGA347983 | 11500972 | - | 4.16E-11 |
| BW16 | 39 | GGaluGA347986 | 11508012 | LOC101751301 | 4.16E-11 |
| BW16 | 39 | Gga_rs14786617 | 11562673 | OSMR | 1.91E-19 |
| BW16 | 39 | GGaluGA348000 | 11576834 | RICTOR | 8.82E-09 |
| BW16 | 39 | Gga_rs14786654 | 11608187 | RICTOR | 8.82E-09 |
| BW16 | 39 | Gga_rs16781411 | 11673306 | FYB | 1.91E-19 |
| BW16 | 39 | Gga_rs14786725 | 11693740 | FYB | 1.91E-19 |
| BW16 | 39 | Gga_rs14786812 | 11788319 | - | 1.91E-19 |
| BW16 | 39 | Gga_rs14755855 | 12043692 | - | 1.91E-19 |
| BW16 | 39 | Gga_rs14755900 | 12085507 | - | 6.62E-39 |
| BW16 | 39 | Gga_rs16102386 | 12129543 | - | 4.92E-10 |
| BW16 | 39 | Gga_rs14755968 | 12151053 | - | 6.62E-39 |
| BW16 | 39 | GGaluGA348083 | 12205308 | - | 4.92E-10 |
| BW16 | 39 | Gga_rs13817336 | 12238730 | LOC101751864 | 4.92E-10 |
| BW16 | 39 | Gga_rs14756044 | 12262828 | PTGER4 | 6.62E-39 |
| BW16 | 39 | Gga_rs14756128 | 12347485 | - | 6.62E-39 |
| BW16 | 39 | Gga_rs14756165 | 12368483 | C7 | 6.62E-39 |
| BW16 | 39 | Gga_rs14756180 | 12389747 | C6 | 1.91E-19 |
| BW16 | 39 | Gga_rs14756234 | 12462388 | LOC100857889 | 6.62E-39 |
| BW16 | 39 | Gga_rs14756256 | 12479404 | LOC100857889 | 6.62E-39 |
| BW16 | 39 | Gga_rs14756284 | 12507150 | LOC100857889 | 6.62E-39 |
| BW16 | 39 | Gga_rs16102951 | 12906327 | - | 1.72E-12 |
| BW16 | 39 | Gga_rs14756703 | 12984581 | - | 1.72E-12 |
| BW16 | 39 | Gga_rs16763012 | 13179849 | NNT | 1.02E-08 |
| BW16 | 39 | Gga_rs14756923 | 13202902 | NNT | 1.02E-08 |
| BW16 | 39 | Gga_rs14756962 | 13221684 | - | 1.06E-10 |
| BW16 | 39 | Gga_rs14757000 | 13288165 | - | 1.02E-08 |
| BW16 | 39 | Gga_rs14757044 | 13376137 | - | 3.92E-09 |
| BW16 | 39 | Gga_rs16763062 | 13447769 | FGF10 | 1.02E-08 |
| BW16 | 39 | Gga_rs16103343 | 13591394 | - | 1.02E-08 |
| BW16 | 39 | GGaluGA348247 | 13611640 | - | 1.02E-08 |
| BW16 | 39 | Gga_rs13728807 | 13707137 | - | 4.42E-12 |
| BW16 | 39 | Gga_rs14757246 | 13714920 | - | 4.42E-12 |
| BW16 | 39 | Gga_rs14757289 | 13794465 | - | 4.42E-12 |
| BW16 | 39 | Gga_rs14757292 | 13812082 | - | 5.09E-09 |
| BW16 | 39 | Gga_rs16103436 | 13835031 | HCN1 | 4.42E-12 |
| BW16 | 39 | Gga_rs14757339 | 13977936 | HCN1 | 1.25E-16 |
| BW16 | 39 | Gga_rs14757367 | 14020461 | HCN1 | 8.54E-11 |
| BW16 | 39 | GGaluGA348291 | 14030756 | HCN1 | 1.25E-16 |
| BW16 | 39 | Gga_rs13728832 | 14084602 | - | 1.25E-16 |
| BW16 | 39 | Gga_rs16103602 | 14189346 | LOC101748367 | 1.73E-31 |
| BW16 | 39 | Gga_rs16763234 | 14205194 | - | 3.14E-16 |
| BW16 | 39 | Gga_rs13728859 | 14260857 | PARP8 | 2.05E-15 |
| BW16 | 39 | GGaluGA348328 | 14328915 | - | 3.45E-15 |
| BW16 | 39 | Gga_rs16103781 | 14444488 | - | 3.45E-15 |
| BW16 | 39 | Gga_rs14757660 | 14468384 | - | 2.64E-09 |
| BW16 | 39 | Gga_rs16103818 | 14584514 | - | 4.69E-09 |
| BW16 | 39 | Gga_rs14757750 | 14738731 | - | 4.69E-09 |
| BW16 | 39 | Gga_rs14757759 | 14765320 | - | 4.74E-07 |
| BW16 | 39 | Gga_rs16103911 | 14851712 | - | 4.69E-09 |
| BW16 | 39 | Gga_rs14757836 | 14876950 | - | 4.69E-09 |
| BW16 | 39 | Gga_rs14757875 | 15142140 | ITGA1 | 4.74E-07 |
| BW16 | 39 | Gga_rs16763572 | 15196080 | ITGA2 | 2.60E-29 |
| BW16 | 39 | Gga_rs16103980 | 15244465 | ITGA2 | 4.69E-09 |
| BW16 | 39 | Gga_rs14757928 | 15257934 | MOCS2 | 4.69E-09 |
| BW16 | 39 | Gga_rs16104128 | 15674108 | ARL15 | 8.01E-11 |
| BW16 | 39 | Gga_rs14758352 | 15898298 | LOC425372 | 2.92E-07 |
| BW16 | 39 | Gga_rs14740460 | 15956247 | - | 2.92E-07 |
| BW16 | 39 | Gga_rs14752031 | 16190785 | PPAP2A | 8.01E-11 |
| BW16 | 39 | GGaluGA348537 | 16520460 | - | 1.87E-06 |
| BW16 | 39 | Gga_rs13676590 | 16867867 | - | 1.90E-22 |
| BW16 | 39 | Gga_rs14752442 | 16892229 | GPBP1 | 1.93E-09 |
| BW16 | 39 | Gga_rs16760371 | 16912728 | GPBP1 | 2.69E-09 |
| BW16 | 39 | Gga_rs14752792 | 17445314 | RAB3C | 1.02E-19 |
| BW16 | 39 | Gga_rs14752843 | 17500236 | RAB3C | 1.93E-09 |
| BW16 | 39 | GGaluGA348716 | 17529244 | RAB3C | 1.93E-09 |
| BW16 | 39 | Gga_rs14752927 | 17612828 | PDE4D | 1.02E-19 |
| BW16 | 39 | GGaluGA348733 | 17687145 | PDE4D | 1.02E-19 |
| BW16 | 39 | Gga_rs14752983 | 17739555 | PDE4D | 7.80E-20 |
| BW16 | 39 | GGaluGA348796 | 18278538 | - | 2.07E-09 |
| BW16 | 39 | GGaluGA348802 | 18308486 | ERCC8 | 2.07E-09 |
| BW16 | 39 | Gga_rs14753237 | 18354247 | NDUFAF2 | 2.07E-09 |
| BW16 | 39 | GGaluGA348847 | 18577655 | ZSWIM6 | 1.25E-16 |
| BW16 | 39 | GGaluGA348898 | 18816948 | KIF2A | 5.96E-16 |
| BW16 | 39 | Gga_rs16100247 | 19021102 | - | 6.00E-08 |
| BW16 | 39 | GGaluGA348948 | 19186417 | - | 5.96E-16 |
| BW16 | 39 | GGaluGA348980 | 19286706 | - | 5.96E-16 |
| BW16 | 39 | GGaluGA348985 | 19313055 | - | 6.00E-08 |
| BW16 | 39 | Gga_rs16100722 | 19834059 | ADAMTS6 | 1.90E-07 |
| BW16 | 39 | GGaluGA349087 | 19992764 | PPWD1 | 8.00E-15 |
| BW16 | 39 | Gga_rs14754344 | 20015776 | TRIM23 | 8.00E-15 |
| BW16 | 39 | Gga_rs13676932 | 20151804 | ERBB2IP | 1.90E-07 |
| BW16 | 39 | Gga_rs14754805 | 20487433 | MAST4 | 2.27E-07 |
| BW16 | 39 | Gga_rs16101219 | 20553419 | MAST4 | 1.39E-14 |
| BW16 | 39 | GGaluGA349195 | 20702054 | MAST4 | 5.43E-07 |
| BW16 | 39 | Gga_rs13677070 | 21139710 | - | 6.11E-12 |
| BW16 | 39 | GGaluGA349294 | 21146543 | - | 6.11E-12 |
| BW16 | 39 | Gga_rs14755437 | 21359710 | CDK7 | 9.79E-07 |
| BW16 | 39 | GGaluGA349348 | 21363270 | CDK7 | 9.79E-07 |
| BW16 | 39 | GGaluGA349353 | 21409504 | SERINC5 | 9.79E-07 |
| BW16 | 39 | GGaluGA349472 | 21899444 | DMGDH | 4.45E-06 |
| BW16 | 39 | GGaluGA349476 | 21909031 | - | 4.81E-06 |
| BW16 | 39 | GGaluGA349488 | 21954151 | ARSB | 2.85E-06 |
| BW1612 | 39 | GGaluGA347594 | 8872779 | PDZD2 | 3.70E-06 |
| BW1612 | 39 | Gga_rs14784649 | 8899679 | PDZD2 | 3.70E-06 |
| BW1612 | 39 | Gga_rs16129521 | 8922444 | PDZD2 | 4.84E-06 |
| BW1612 | 39 | Gga_rs16129626 | 9088090 | MTMR12 | 1.92E-06 |
| BW1612 | 39 | GGaluGA347637 | 9095810 | - | 3.67E-06 |
| BW1612 | 39 | Gga_rs14784876 | 9131966 | ZFR | 3.67E-06 |
| BW1612 | 39 | GGaluGA347642 | 9146754 | - | 3.67E-06 |
| BW1612 | 39 | Gga_rs13817660 | 11113817 | GDNF | 1.59E-07 |
| BW1612 | 39 | Gga_rs13800656 | 11327074 | EGFLAM | 2.68E-08 |
| BW1612 | 39 | GGaluGA347965 | 11370132 | EGFLAM | 2.68E-08 |
| BW1612 | 39 | Gga_rs14755900 | 12085507 | - | 3.91E-08 |
| BW1612 | 39 | Gga_rs14755968 | 12151053 | - | 3.91E-08 |
| BW1612 | 39 | Gga_rs14756044 | 12262828 | PTGER4 | 3.91E-08 |
| BW1612 | 39 | Gga_rs14756128 | 12347485 | - | 3.91E-08 |
| BW1612 | 39 | Gga_rs14756165 | 12368483 | C7 | 3.91E-08 |
| BW1612 | 39 | Gga_rs14756234 | 12462388 | LOC100857889 | 3.91E-08 |
| BW1612 | 39 | Gga_rs14756256 | 12479404 | LOC100857889 | 3.91E-08 |
| BW1612 | 39 | Gga_rs14756284 | 12507150 | LOC100857889 | 3.91E-08 |
| BW1612 | 39 | Gga_rs16103602 | 14189346 | LOC101748367 | 9.74E-07 |
| BW1612 | 39 | Gga_rs16763572 | 15196080 | ITGA2 | 3.23E-06 |
| SL08 | 39 | Gga_rs14066883 | 2937208 | - | 2.19E-06 |
| SL08 | 39 | Gga_rs16455373 | 3376839 | RIT2 | 2.30E-06 |
| SL08 | 39 | Gga_rs16455433 | 3447887 | - | 2.30E-06 |
| SL08 | 39 | Gga_rs16455310 | 3580256 | - | 2.30E-06 |
| SL08 | 39 | Gga_rs14507133 | 3829236 | - | 7.65E-07 |
| SL08 | 39 | Gga_rs14507111 | 3875165 | LOC101751865 | 8.91E-07 |
| SL08 | 39 | Gga_rs14698404 | 4371086 | - | 4.64E-06 |
| SL08 | 39 | Gga_rs14736673 | 4495800 | - | 4.16E-06 |
| SL08 | 39 | Gga_rs14695654 | 4646914 | - | 6.52E-08 |
| SL08 | 39 | Gga_rs13781756 | 5683707 | CELF4 | 6.79E-08 |
| SL08 | 39 | Gga_rs16687179 | 5909968 | CELF4 | 6.79E-08 |
| SL08 | 39 | Gga_rs14689250 | 5980966 | CELF4 | 6.79E-08 |
| SL08 | 39 | Gga_rs14689275 | 6023957 | CELF4 | 5.40E-08 |
| SL08 | 39 | Gga_rs14785130 | 9581584 | ADAMTS12 | 3.32E-06 |
| SL08 | 39 | Gga_rs14785179 | 9659034 | ADAMTS12 | 3.52E-06 |
| SL08 | 39 | Gga_rs14785203 | 9700700 | - | 3.52E-06 |
| SL08 | 39 | Gga_rs14785235 | 9744903 | - | 1.88E-06 |
| SL08 | 39 | Gga_rs14708266 | 9808980 | - | 3.40E-07 |
| SL08 | 39 | Gga_rs16130332 | 9833681 | RAI14 | 5.90E-07 |
| SL08 | 39 | Gga_rs16780726 | 9871880 | RAI14 | 5.90E-07 |
| SL08 | 39 | Gga_rs14785443 | 9975736 | - | 9.23E-07 |
| SL08 | 39 | GGaluGA347787 | 9995191 | - | 9.23E-07 |
| SL08 | 39 | Gga_rs16130424 | 10049151 | - | 9.23E-07 |
| SL08 | 39 | Gga_rs16780806 | 10065018 | - | 8.81E-07 |
| SL08 | 39 | Gga_rs14731045 | 10187477 | SPEF2 | 6.61E-07 |
| SL08 | 39 | Gga_rs14731057 | 10212209 | - | 6.61E-07 |
| SL08 | 39 | Gga_rs16067654 | 10234193 | IL7R | 6.61E-07 |
| SL08 | 39 | GGaluGA347845 | 10361382 | NADKD1 | 1.10E-06 |
| SL08 | 39 | Gga_rs13817561 | 10463085 | - | 1.19E-07 |
| SL08 | 39 | Gga_rs14785793 | 10575355 | SLC1A3 | 1.19E-07 |
| SL08 | 39 | GGaluGA347920 | 10983124 | WDR70 | 8.23E-11 |
| SL08 | 39 | Gga_rs13817660 | 11113817 | GDNF | 1.28E-17 |
| SL08 | 39 | Gga_rs14786270 | 11214452 | - | 4.83E-06 |
| SL08 | 39 | Gga_rs13800656 | 11327074 | EGFLAM | 3.57E-42 |
| SL08 | 39 | GGaluGA347965 | 11370132 | EGFLAM | 3.57E-42 |
| SL08 | 39 | Gga_rs14786514 | 11472405 | - | 1.92E-20 |
| SL08 | 39 | GGaluGA347983 | 11500972 | - | 1.10E-10 |
| SL08 | 39 | GGaluGA347986 | 11508012 | LOC101751301 | 1.10E-10 |
| SL08 | 39 | Gga_rs14786617 | 11562673 | OSMR | 5.33E-22 |
| SL08 | 39 | GGaluGA348000 | 11576834 | RICTOR | 1.20E-08 |
| SL08 | 39 | Gga_rs14786654 | 11608187 | RICTOR | 1.20E-08 |
| SL08 | 39 | Gga_rs16781411 | 11673306 | FYB | 5.33E-22 |
| SL08 | 39 | Gga_rs14786725 | 11693740 | FYB | 5.33E-22 |
| SL08 | 39 | Gga_rs14786812 | 11788319 | - | 5.33E-22 |
| SL08 | 39 | Gga_rs14755855 | 12043692 | - | 5.33E-22 |
| SL08 | 39 | Gga_rs14755900 | 12085507 | - | 5.10E-45 |
| SL08 | 39 | Gga_rs16102386 | 12129543 | - | 1.60E-08 |
| SL08 | 39 | Gga_rs14755968 | 12151053 | - | 5.10E-45 |
| SL08 | 39 | GGaluGA348083 | 12205308 | - | 1.60E-08 |
| SL08 | 39 | Gga_rs13817336 | 12238730 | LOC101751864 | 1.60E-08 |
| SL08 | 39 | Gga_rs14756044 | 12262828 | PTGER4 | 5.10E-45 |
| SL08 | 39 | Gga_rs14756128 | 12347485 | - | 5.10E-45 |
| SL08 | 39 | Gga_rs14756165 | 12368483 | C7 | 5.10E-45 |
| SL08 | 39 | Gga_rs14756180 | 12389747 | C6 | 5.33E-22 |
| SL08 | 39 | Gga_rs14756234 | 12462388 | LOC100857889 | 5.10E-45 |
| SL08 | 39 | Gga_rs14756256 | 12479404 | LOC100857889 | 5.10E-45 |
| SL08 | 39 | Gga_rs14756284 | 12507150 | LOC100857889 | 5.10E-45 |
| SL08 | 39 | Gga_rs16102951 | 12906327 | - | 2.19E-12 |
| SL08 | 39 | Gga_rs14756703 | 12984581 | - | 2.19E-12 |
| SL08 | 39 | Gga_rs16763012 | 13179849 | NNT | 4.39E-11 |
| SL08 | 39 | Gga_rs14756923 | 13202902 | NNT | 4.39E-11 |
| SL08 | 39 | Gga_rs14756962 | 13221684 | - | 1.66E-09 |
| SL08 | 39 | Gga_rs14757000 | 13288165 | - | 4.39E-11 |
| SL08 | 39 | Gga_rs14757044 | 13376137 | - | 3.08E-08 |
| SL08 | 39 | Gga_rs16763062 | 13447769 | FGF10 | 4.39E-11 |
| SL08 | 39 | Gga_rs16103343 | 13591394 | - | 4.39E-11 |
| SL08 | 39 | GGaluGA348247 | 13611640 | - | 4.39E-11 |
| SL08 | 39 | Gga_rs13728807 | 13707137 | - | 2.66E-12 |
| SL08 | 39 | Gga_rs14757246 | 13714920 | - | 2.66E-12 |
| SL08 | 39 | Gga_rs14757289 | 13794465 | - | 2.66E-12 |
| SL08 | 39 | Gga_rs14757292 | 13812082 | - | 1.59E-08 |
| SL08 | 39 | Gga_rs16103436 | 13835031 | HCN1 | 2.66E-12 |
| SL08 | 39 | Gga_rs14757339 | 13977936 | HCN1 | 2.74E-19 |
| SL08 | 39 | Gga_rs14757367 | 14020461 | HCN1 | 5.78E-11 |
| SL08 | 39 | GGaluGA348291 | 14030756 | HCN1 | 2.74E-19 |
| SL08 | 39 | Gga_rs13728832 | 14084602 | - | 2.74E-19 |
| SL08 | 39 | Gga_rs16103602 | 14189346 | LOC101748367 | 1.70E-34 |
| SL08 | 39 | Gga_rs16763234 | 14205194 | - | 3.96E-18 |
| SL08 | 39 | Gga_rs13728859 | 14260857 | PARP8 | 2.41E-17 |
| SL08 | 39 | GGaluGA348328 | 14328915 | - | 6.05E-17 |
| SL08 | 39 | Gga_rs16103781 | 14444488 | - | 6.05E-17 |
| SL08 | 39 | Gga_rs14757660 | 14468384 | - | 1.42E-08 |
| SL08 | 39 | Gga_rs16103818 | 14584514 | - | 2.40E-07 |
| SL08 | 39 | Gga_rs16763394 | 14658324 | - | 4.22E-07 |
| SL08 | 39 | Gga_rs14757750 | 14738731 | - | 2.40E-07 |
| SL08 | 39 | Gga_rs16103911 | 14851712 | - | 2.40E-07 |
| SL08 | 39 | Gga_rs14757836 | 14876950 | - | 2.40E-07 |
| SL08 | 39 | Gga_rs16763572 | 15196080 | ITGA2 | 2.44E-32 |
| SL08 | 39 | Gga_rs16763589 | 15222404 | ITGA2 | 4.22E-07 |
| SL08 | 39 | Gga_rs16103980 | 15244465 | ITGA2 | 2.40E-07 |
| SL08 | 39 | Gga_rs14757928 | 15257934 | MOCS2 | 2.40E-07 |
| SL08 | 39 | Gga_rs16104128 | 15674108 | ARL15 | 9.33E-13 |
| SL08 | 39 | Gga_rs14758352 | 15898298 | LOC425372 | 1.41E-09 |
| SL08 | 39 | Gga_rs14740460 | 15956247 | - | 1.41E-09 |
| SL08 | 39 | Gga_rs14752031 | 16190785 | PPAP2A | 9.33E-13 |
| SL08 | 39 | GGaluGA348537 | 16520460 | - | 4.66E-08 |
| SL08 | 39 | Gga_rs14752383 | 16798487 | MAP3K1 | 4.85E-07 |
| SL08 | 39 | Gga_rs13676590 | 16867867 | - | 3.18E-25 |
| SL08 | 39 | Gga_rs14752442 | 16892229 | GPBP1 | 4.56E-11 |
| SL08 | 39 | Gga_rs16760371 | 16912728 | GPBP1 | 3.95E-11 |
| SL08 | 39 | Gga_rs13676607 | 16958770 | - | 9.99E-07 |
| SL08 | 39 | Gga_rs14752792 | 17445314 | RAB3C | 8.29E-23 |
| SL08 | 39 | Gga_rs14752843 | 17500236 | RAB3C | 4.56E-11 |
| SL08 | 39 | GGaluGA348716 | 17529244 | RAB3C | 4.56E-11 |
| SL08 | 39 | Gga_rs14752927 | 17612828 | PDE4D | 8.29E-23 |
| SL08 | 39 | GGaluGA348733 | 17687145 | PDE4D | 8.29E-23 |
| SL08 | 39 | Gga_rs14752983 | 17739555 | PDE4D | 8.40E-23 |
| SL08 | 39 | GGaluGA348796 | 18278538 | - | 1.31E-10 |
| SL08 | 39 | GGaluGA348802 | 18308486 | ERCC8 | 1.31E-10 |
| SL08 | 39 | Gga_rs14753237 | 18354247 | NDUFAF2 | 1.31E-10 |
| SL08 | 39 | GGaluGA348847 | 18577655 | ZSWIM6 | 6.35E-20 |
| SL08 | 39 | GGaluGA348898 | 18816948 | KIF2A | 5.36E-19 |
| SL08 | 39 | Gga_rs16100247 | 19021102 | - | 1.25E-09 |
| SL08 | 39 | GGaluGA348948 | 19186417 | - | 5.36E-19 |
| SL08 | 39 | GGaluGA348980 | 19286706 | - | 5.36E-19 |
| SL08 | 39 | GGaluGA348985 | 19313055 | - | 1.25E-09 |
| SL08 | 39 | Gga_rs16100722 | 19834059 | ADAMTS6 | 3.27E-09 |
| SL08 | 39 | GGaluGA349087 | 19992764 | PPWD1 | 6.37E-18 |
| SL08 | 39 | Gga_rs14754344 | 20015776 | TRIM23 | 6.37E-18 |
| SL08 | 39 | Gga_rs13676932 | 20151804 | ERBB2IP | 3.27E-09 |
| SL08 | 39 | Gga_rs14754805 | 20487433 | MAST4 | 4.96E-09 |
| SL08 | 39 | Gga_rs16101219 | 20553419 | MAST4 | 4.56E-17 |
| SL08 | 39 | GGaluGA349195 | 20702054 | MAST4 | 1.05E-08 |
| SL08 | 39 | Gga_rs13677070 | 21139710 | - | 1.38E-13 |
| SL08 | 39 | GGaluGA349294 | 21146543 | - | 1.38E-13 |
| SL08 | 39 | Gga_rs14755437 | 21359710 | CDK7 | 7.90E-09 |
| SL08 | 39 | GGaluGA349348 | 21363270 | CDK7 | 7.90E-09 |
| SL08 | 39 | GGaluGA349353 | 21409504 | SERINC5 | 7.90E-09 |
| SL08 | 39 | GGaluGA349472 | 21899444 | DMGDH | 5.12E-07 |
| SL08 | 39 | GGaluGA349476 | 21909031 | - | 2.14E-07 |
| SL08 | 39 | GGaluGA349488 | 21954151 | ARSB | 6.96E-07 |
| SRBC14 | 14 | Gga_rs10724420 | 7418129 | PDPK1 | 1.21E-06 |
| GGA: gallus gallus chromosome; Pos (bp): positon (base pair). | | | | | |
