# Supplementary material for: Detection of QTL for traits related to adaptation to sub-optimal climatic conditions in chickens
Source: Genet Sel Evol. 2017 Apr 20;49:39. doi: 10.1186/s12711-017-0314-5 (PMC5399330; doi:10.1186/s12711-017-0314-5)
Supplement: Supplementary file 8 — Additional file 8: Table S3. Results of the whole-genome sequence analysis: list of genes showing SNPs between the L2 and the R- birds in the genome-wide QTL regions detected by either QTLMap or GEMMA. SNPs were classified according to their position in the gene. [file 12711_2017_314_MOESM8_ESM.docx]

Table S3 Results of the whole genome sequence analysis: list of genes showing SNP variants between the L2 and the R- birds in the genome-wide QTL regions detected by either QTLMap or GEMMA. SNP variants were classified according to their position in the gene

| **Trait** | **QTL region** | **Gene Symbol** | **Total Nb. of SNP** | **Nb. of known SNP** | **Nb. of new SNP** | **Nb. of 3' UTR** | **Nb. of 5' UTR** | **Nb. of downstream SNP** | **Nb. of intron** | **Nb. of missense** | **Nb. of upstream** |
| --- | --- | --- | --- | --- | --- | --- | --- | --- | --- | --- | --- |
| BW00 | 1:136911762-137765611 | ATP11A | 442 | 431 | 11 | 0 | 0 | 0 | 4 | 0 | 7 |
| BW00 | 1:136911762-137765611 | F10 | 34 | 29 | 5 | 0 | 0 | 0 | 0 | 0 | 5 |
| BW00 | 1:136911762-137765611 | F7 | 71 | 65 | 6 | 0 | 0 | 5 | 0 | 0 | 1 |
| BW00 | 1:136911762-137765611 | MCF2L | 480 | 468 | 12 | 0 | 0 | 1 | 10 | 0 | 0 |
| BW00 | 1:136911762-137765611 | MYO16 | 923 | 879 | 44 | 0 | 0 | 0 | 43 | 0 | 1 |
| BW00 | 1:136911762-137765611 | PROZ | 4 | 4 | 0 | 0 | 0 | 0 | 0 | 0 | 0 |
| BW00 | 1:136911762-137765611 | TUBGCP3 | 251 | 229 | 22 | 0 | 0 | 1 | 21 | 0 | 0 |
| BW16 | 2:111216288-112068091 | CYP7A1 | 106 | 80 | 26 | 0 | 0 | 5 | 9 | 0 | 12 |
| BW16 | 2:111216288-112068091 | FAM110B | 45 | 37 | 8 | 0 | 0 | 3 | 0 | 0 | 5 |
| BW16 | 2:111216288-112068091 | IMPAD1 | 100 | 71 | 29 | 0 | 0 | 0 | 13 | 0 | 16 |
| BW16 | 2:111216288-112068091 | NSMAF | 273 | 257 | 16 | 0 | 0 | 4 | 11 | 0 | 0 |
| BW16 | 2:111216288-112068091 | SDCBP | 110 | 92 | 18 | 0 | 1 | 1 | 15 | 0 | 1 |
| BW16 | 2:111216288-112068091 | TOX | 446 | 401 | 45 | 1 | 0 | 2 | 42 | 0 | 0 |
| BW16 | 2:111216288-112068091 | UBXN2B | 100 | 86 | 14 | 0 | 1 | 0 | 9 | 0 | 5 |
| BW00 | 2:41103517-42183240 | ABHD5 | 30 | 24 | 6 | 0 | 0 | 0 | 3 | 0 | 3 |
| BW00 | 2:41103517-42183240 | ANO10 | 90 | 88 | 2 | 0 | 0 | 1 | 1 | 0 | 0 |
| BW00 | 2:41103517-42183240 | C2H3ORF39 | 97 | 97 | 0 | 0 | 0 | 0 | 0 | 0 | 0 |
| BW00 | 2:41103517-42183240 | CAPN7 | 18 | 14 | 4 | 0 | 0 | 1 | 3 | 0 | 0 |
| BW00 | 2:41103517-42183240 | CNOT10 | 58 | 54 | 4 | 0 | 0 | 3 | 1 | 0 | 0 |
| BW00 | 2:41103517-42183240 | EAF1 | 26 | 25 | 1 | 0 | 0 | 1 | 0 | 0 | 0 |
| BW00 | 2:41103517-42183240 | ENTPD3 | 86 | 75 | 11 | 0 | 0 | 2 | 8 | 0 | 1 |
| BW00 | 2:41103517-42183240 | FAM198A | 84 | 78 | 6 | 0 | 0 | 2 | 3 | 0 | 1 |
| BW00 | 2:41103517-42183240 | METTL6 | 2 | 2 | 0 | 0 | 0 | 0 | 0 | 0 | 0 |
| BW00 | 2:41103517-42183240 | MTURN | 68 | 64 | 4 | 0 | 0 | 0 | 2 | 0 | 2 |
| BW00 | 2:41103517-42183240 | NOD1 | 48 | 47 | 1 | 0 | 0 | 0 | 0 | 0 | 1 |
| BW00 | 2:41103517-42183240 | RPL14 | 44 | 39 | 5 | 0 | 0 | 1 | 2 | 0 | 2 |
| BW00 | 2:41103517-42183240 | SH3BP5 | 180 | 167 | 13 | 0 | 0 | 0 | 13 | 0 | 0 |
| BW00 | 2:41103517-42183240 | SNRK | 14 | 12 | 2 | 0 | 0 | 0 | 2 | 0 | 0 |
| BW00 | 2:41103517-42183240 | TCAIM | 84 | 83 | 1 | 0 | 0 | 0 | 1 | 0 | 0 |
| BW00 | 2:41103517-42183240 | TOPAZ1 | 207 | 127 | 80 | 0 | 0 | 23 | 52 | 1 | 4 |
| BW00 | 2:41103517-42183240 | TRIM71 | 50 | 37 | 13 | 0 | 0 | 1 | 12 | 0 | 0 |
| BW00 | 2:41103517-42183240 | ZNRF2 | 223 | 222 | 1 | 0 | 0 | 0 | 1 | 0 | 0 |
| BW00 | 4:50414488-51349139 | CENPC | 187 | 179 | 8 | 0 | 0 | 0 | 8 | 0 | 0 |
| BW00 | 4:50414488-51349139 | EPHA5 | 647 | 628 | 19 | 0 | 0 | 0 | 17 | 1 | 1 |
| BW00 | 4:50414488-51349139 | SNOR12 | 10 | 10 | 0 | 0 | 0 | 0 | 0 | 0 | 0 |
| BW00 | 4:50414488-51349139 | STAP1 | 62 | 54 | 8 | 0 | 0 | 4 | 1 | 0 | 3 |
| BW00 | 4:50414488-51349139 | UBA6 | 133 | 99 | 34 | 0 | 0 | 3 | 23 | 0 | 4 |
| BW00 | 9:19678986-20259257 | MECOM | 205 | 185 | 20 | 0 | 0 | 0 | 20 | 0 | 0 |
| BW04 | 14:2823550-3306419 | ACTB | 75 | 63 | 12 | 0 | 0 | 38 | 11 | 0 | 21 |
| BW04 | 14:2823550-3306419 | AP5Z1 | 173 | 153 | 20 | 8 | 0 | 58 | 74 | 4 | 77 |
| BW04 | 14:2823550-3306419 | CHST12 | 86 | 78 | 8 | 1 | 7 | 51 | 6 | 0 | 47 |
| BW04 | 14:2823550-3306419 | CYP3A4 | 84 | 83 | 1 | 0 | 0 | 37 | 25 | 1 | 0 |
| BW04 | 14:2823550-3306419 | CYP3A7 | 173 | 144 | 29 | 2 | 0 | 50 | 85 | 5 | 26 |
| BW04 | 14:2823550-3306419 | EIF3B | 289 | 267 | 22 | 0 | 0 | 21 | 188 | 0 | 16 |
| BW04 | 14:2823550-3306419 | FBXL18 | 164 | 155 | 9 | 1 | 0 | 20 | 105 | 2 | 19 |
| BW04 | 14:2823550-3306419 | FOXK1 | 125 | 117 | 8 | 3 | 0 | 28 | 66 | 0 | 26 |
| BW04 | 14:2823550-3306419 | FSCN1 | 101 | 77 | 24 | 4 | 0 | 18 | 30 | 0 | 61 |
| BW04 | 14:2823550-3306419 | FTSJ2 | 97 | 82 | 15 | 0 | 0 | 29 | 15 | 1 | 45 |
| BW04 | 24:3741253-4004679 | ARHGEF12 | 1231 | 1079 | 152 | 0 | 0 | 0 | 1073 | 0 | 158 |
| BW04 | 24:3741253-4004679 | C6 | 10 | 2 | 8 | 0 | 0 | 0 | 9 | 0 | 1 |
| BW04 | 24:3741253-4004679 | C7 | 12 | 12 | 0 | 0 | 0 | 0 | 0 | 0 | 11 |
| BW04 | 24:3741253-4004679 | DAB2 | 106 | 96 | 10 | 6 | 0 | 29 | 67 | 0 | 9 |
| BW04 | 24:3741253-4004679 | EGFLAM | 472 | 457 | 15 | 2 | 1 | 43 | 410 | 2 | 18 |
| BW00 | 27:4031959-4467930 | CWC25 | 120 | 108 | 12 | 0 | 0 | 3 | 0 | 0 | 9 |
| BW00 | 27:4031959-4467930 | FBXO47 | 113 | 105 | 8 | 0 | 0 | 5 | 0 | 0 | 3 |
| BW00 | 27:4031959-4467930 | LASP1 | 134 | 106 | 28 | 0 | 0 | 6 | 20 | 0 | 2 |
| BW00 | 27:4031959-4467930 | PIP4K2B | 5 | 5 | 0 | 0 | 0 | 0 | 0 | 0 | 0 |
| BW00 | 27:4031959-4467930 | PLXDC1 | 19 | 19 | 0 | 0 | 0 | 0 | 0 | 0 | 0 |
| BW00 | 27:4031959-4467930 | RPL23 | 92 | 69 | 23 | 0 | 0 | 11 | 10 | 0 | 2 |
| BW00 | 27:4031959-4467930 | SNORA21 | 79 | 58 | 21 | 0 | 0 | 11 | 0 | 0 | 10 |
| BW16 | Z:11761478-13036599 | EMB | 88 | 79 | 9 | 14 | 1 | 27 | 32 | 1 | 13 |
| BW16 | Z:11761478-13036599 | EP4 | 106 | 98 | 8 | 0 | 1 | 15 | 52 | 1 | 33 |
| BW16 | Z:11761478-13036599 | FGF10 | 189 | 159 | 30 | 0 | 0 | 12 | 172 | 1 | 4 |
| BW16 | Z:11761478-13036599 | FYB | 260 | 237 | 23 | 12 | 1 | 26 | 210 | 3 | 35 |
| BW16 | Z:11761478-13036599 | HCN1 | 509 | 359 | 150 | 0 | 1 | 3 | 500 | 1 | 0 |
| BW16 | Z:11761478-13036599 | LIFR | 176 | 168 | 8 | 0 | 0 | 11 | 148 | 0 | 14 |
| BW08, BW0804, BW12, SL08 | Z:12073533-13036599 | MRPS30 | 92 | 77 | 15 | 16 | 1 | 36 | 15 | 2 | 31 |
| BW08, BW0804, BW12, SL08 | Z:12073533-13036599 | OAF | 474 | 426 | 48 | 15 | 0 | 90 | 158 | 0 | 207 |
| BW08, BW0804, BW12, SL08 | Z:12073533-13036599 | OSMR | 143 | 136 | 7 | 3 | 4 | 16 | 103 | 1 | 30 |
| BW08, BW0804, BW12, SL08 | Z:12073533-13036599 | OXCT1 | 1 | 0 | 1 | 1 | 0 | 1 | 0 | 0 | 0 |
| BW08, BW0804, BW12, SL08 | Z:12073533-13036599 | PLCXD3 | 31 | 8 | 23 | 0 | 0 | 0 | 26 | 0 | 5 |
| BW08, BW0804, BW12, SL08 | Z:12073533-13036599 | POU2F3 | 671 | 565 | 106 | 6 | 0 | 83 | 498 | 0 | 80 |
| BW08, BW0804, BW12, SL08 | Z:12073533-13036599 | PRKAA1 | 149 | 141 | 8 | 10 | 1 | 47 | 59 | 0 | 30 |
| BW08, BW0804, BW12, SL08 | Z:12073533-13036599 | PTGER4 | 104 | 96 | 8 | 0 | 1 | 15 | 52 | 1 | 31 |
| BW08, BW0804, BW12, SL08 | Z:12073533-13036599 | RICTOR | 195 | 180 | 15 | 0 | 0 | 0 | 154 | 0 | 41 |
| BW04 | Z:13665778-14652110 | RPL37 | 103 | 97 | 6 | 1 | 1 | 30 | 22 | 0 | 49 |
| BW04 | Z:13665778-14652110 | SNORD72 | 78 | 74 | 4 | 0 | 0 | 30 | 0 | 0 | 48 |
| BW04 | Z:13665778-14652110 | TMEM136 | 289 | 253 | 36 | 27 | 4 | 99 | 35 | 3 | 118 |
| BW04 | Z:13665778-14652110 | TMEM136-1 | 99 | 92 | 7 | 10 | 0 | 39 | 8 | 0 | 39 |
| BW04 | Z:13665778-14652110 | TRIM29 | 409 | 365 | 44 | 34 | 2 | 67 | 196 | 0 | 100 |
| BW04 | Z:13665778-14652110 | TTC33 | 256 | 223 | 33 | 0 | 0 | 26 | 203 | 0 | 26 |
| BW04 | Z:13665778-14652110 | ZOV3 | 75 | 67 | 8 | 14 | 0 | 27 | 18 | 1 | 15 |
